# NFκB and JNK pathways mediate metabolic adaptation upon ESCRT-I deficiency

**DOI:** 10.1007/s00018-024-05490-y

**Published:** 2024-11-19

**Authors:** Jaroslaw Cendrowski, Marta Wrobel, Michal Mazur, Bartosz Jary, Ranjana Maurya, Surui Wang, Michal Korostynski, Anna Dziewulska, Maria Rohm, Patryk Kuropka, Natalia Pudelko-Malik, Piotr Mlynarz, Agnieszka Dobrzyn, Anja Zeigerer, Marta Miaczynska

**Affiliations:** 1 Laboratory of Cell Biology, International Institute of Molecular and Cell Biology, Warsaw, Poland; 2 Institute for Diabetes and Cancer, Helmholtz Munich, Neuherberg, Germany; 3 Joint Heidelberg-IDC Translational Diabetes Program, Inner Medicine 1, University Hospital, Heidelberg, Germany; 4 German Center for Diabetes Research, Neuherberg, Germany; 5 Laboratory of Pharmacogenomics, Department of Molecular Neuropharmacology, Institute of Pharmacology Polish Academy of Sciences, Krakow, Poland; 6 Laboratory of Cell Signaling and Metabolic Disorders, Nencki Institute of Experimental Biology, Warsaw, Poland; 7 Department of Biochemistry, Molecular Biology and Biotechnology, Faculty of Chemistry, Wroclaw University of Science and Technology, Wroclaw, Poland; 8 European Center for Angioscience (ECAS), Medical Faculty Mannheim, Heidelberg University, Mannheim, Germany; 9 Present Address: Department of Genetics, Maria Sklodowska-Curie National Research Institute of Oncology, Warsaw, Poland

**Keywords:** ESCRT, glycolysis, fatty acid oxidation, mitochondria, NFκB, JNK

## Abstract

Endosomal Sorting Complexes Required for Transport (ESCRTs) are crucial for delivering membrane receptors or intracellular organelles for lysosomal degradation which provides the cell with lysosome-derived nutrients. Yet, how ESCRT dysfunction affects cell metabolism remained elusive. To address this, we analyzed transcriptomes of cells lacking TSG101 or VPS28 proteins, components of ESCRT-I subcomplex. ESCRT-I deficiency reduced the expression of genes encoding enzymes involved in oxidation of fatty acids and amino acids, such as branched-chain amino acids, and increased the expression of genes encoding glycolytic enzymes. The changes in metabolic gene expression were associated with Warburg effect-like metabolic reprogramming that included intracellular accumulation of lipids, increased glucose/glutamine consumption and lactate production. Moreover, depletion of ESCRT-I components led to expansion of the ER and accumulation of small mitochondria, most of which retained proper potential and performed ATP-linked respiration. Mechanistically, the observed transcriptional reprogramming towards glycolysis in the absence of ESCRT-I occurred due to activation of the canonical NFκB and JNK signaling pathways and at least in part by perturbed lysosomal degradation. We propose that by activating the stress signaling pathways ESCRT-I deficiency leads to preferential usage of extracellular nutrients, like glucose and glutamine, for energy production instead of lysosome-derived nutrients, such as fatty acids and branched-chain amino acids.

**Supplementary Information:**

The online version contains supplementary material available at 10.1007/s00018-024-05490-y.

## Introduction

In order to generate energy, mammalian cells metabolize nutrients, such as glucose, free fatty acids (FFAs) or amino acids, including branched chain amino acids (BCAAs). Typically, these nutrients undergo oxidative metabolism, with initial reactions leading to generation of esters of coenzyme A (CoA), for instance acetylated coenzyme A (acetyl-CoA). These intermediate metabolites are subsequently introduced into the citric acid cycle in mitochondria. Whereas glucose oxidation is often the major source of energy to support cell growth and function [1], in some physiological or pathological conditions cells may prefer to oxidize FFAs or BCAAs [2]. For instance, oxidative breakdown of FFAs, known as β-oxidation, is a crucial energy source when glucose supply is limited [3].

Some cell types rely on oxygen-independent phase of glucose metabolism, glycolysis. Glycolysis is the initial chain of reactions of glucose breakdown that occurs in the cytoplasm and leads to generation of pyruvate [1]. In cells relying on glycolysis, such as stem cells [4], pyruvate instead of being transformed into acetyl-CoA is converted in the cytosol into lactate, an acidic metabolite that may be released outside the cells to avoid its toxicity [1]. Certain conditions may cause changes in cellular bioenergetics affecting preference for oxidative or glycolytic metabolism, termed metabolic reprogramming. Such reprogramming occurs often in cancer cells (known as the Warburg effect) that utilize glycolytic metabolism even in the presence of oxygen (aerobic glycolysis) instead of oxidative metabolism [5, 6]. Another example is inflammation upon which metabolic reprogramming is associated with increased aerobic glycolysis, inhibition of β-oxidation and intracellular accumulation of lipids [7, 8]**.** Although glycolysis is less efficient in ATP production than oxidative glucose metabolism, recent studies have shown that cancer cells that exert the Warburg effect do not suffer from ATP deficiency, as they use glutamine as a source of mitochondria-derived energy [9]. Moreover, these cells use glutamine for fatty acid synthesis [10] and other amino acids, serine and glycine, for fueling the one-carbon metabolism that promotes biosynthesis of nucleotides and anti-oxidative molecules, including glutathione [11].

Increasing evidence indicates that cellular metabolism strongly depends on the function of lysosomes [12, 13], organelles that degrade different types of cargo delivered by various membrane transport pathways [14]. For instance, lysosomes receive external macromolecules and plasma membrane proteins via endocytosis (endolysosomal degradation) whereas intracellular cargo including protein aggregates or damaged organelles through macroautophagy (autolysosomal degradation). Endolysosomal degradation of membrane receptors restricts their signaling [15] and this may affect intracellular metabolic cues [16]. Autolysosomal degradation controls the turnover and hence the abundance of metabolic organelles such as mitochondria [17]. Lysosomes are an important intracellular source of nutrients including fatty acids or amino acids that come from lysosomal degradation of lipids or proteins [18]. The provision of lysosome-derived nutrients to the cell is sensed by lysosome-associated signaling pathways [19], including signaling of mechanistic Target of Rapamycin Complex 1 (mTORC1) that is activated by lysosome-derived amino acids or cholesterol to promote anabolic and inhibit catabolic pathways [20, 21].

Endosomal Sorting Complexes Required for Transport (ESCRTs) may be particularly implicated in regulation of cell metabolism by lysosomes. ESCRTs encompass several protein assemblies (ESCRT-0, I, II, and III) that mediate membrane remodeling in endocytosis, autophagy, cytokinesis, nuclear envelope sealing, and virus budding [22]. Owing to some of their functions, ESCRTs fuel lysosomes with cargo from multiple sources [23], for instance by participating in endolysosomal degradation of many signaling receptors [24], in autolysosomal clearance of protein aggregates [25] and in endolysosomal or autolysosomal degradation of mitochondria [26, 27].

ESCRT dysfunction leads to activation of some stress response pathways (reviewed in [24, 28]). As we previously described, in mammalian cells, depletion of ESCRT-I components impairs endolysosomal degradation of cytokine receptors [29, 30]. Their endosomal accumulation leads to activation of canonical and non-canonical NFκB signaling, hallmarked by phosphorylation of RELA and accumulation of p52 transcription factors respectively, and induced expression of inflammatory genes [29–31]. Others have shown that ESCRT dysfunction may cause activation of JNK signaling [32, 33]. Canonical NFκB signaling was found to inhibit fatty acid oxidation during cardiac hypertrophy [34] and to promote aerobic glycolysis in sarcoma cells [35], whereas JNK signaling was reported to either inhibit or promote glycolytic metabolism [36]. However, whether the regulation of NFκB or JNK pathways upon ESCRT dysfunction has consequences for cell metabolism has not been addressed.

Our recent studies began to unravel particular metabolic pathways regulated by ESCRT. VPS37A, one of ESCRT-I components was shown to have a specific function in the liver in mediating glucagon receptor degradation, thereby regulating hepatic glucose production [37]. In another study we discovered that by fueling lysosomes with macromolecules from multiple sources, ESCRT-I promotes substrate-specific mTORC1 signaling that inhibits TFEB/TFE3 transcription factors [14]. While ESCRT-I depletion does not affect the general mTORC1 signaling, it leads to activation of TFEB/TFE3 transcription factors that stimulate the expression of genes involved in lysosome biogenesis [14]. Although TFEB/TFE3 signaling may control lipid metabolism and mitochondria biogenesis [38, 39], it remains unknown whether activation of these transcription factors affects cell metabolism upon ESCRT deficiency.

Here, we aimed at studying metabolic consequences of ESCRT-I deficiency. By a transcriptomic approach and its subsequent validation, we discovered that ESCRT-I depletion causes a transcriptional reprogramming towards glycolytic metabolism that occurs due to activation of NFκB and JNK signaling pathways and likely as a result of inhibited lysosomal degradation.

## Results

### Lack of ESCRT-I leads to reduced expression of genes involved in oxidative metabolism of carboxylic acid-containing molecules such as amino acids and fatty acids

**Fig. 1:**
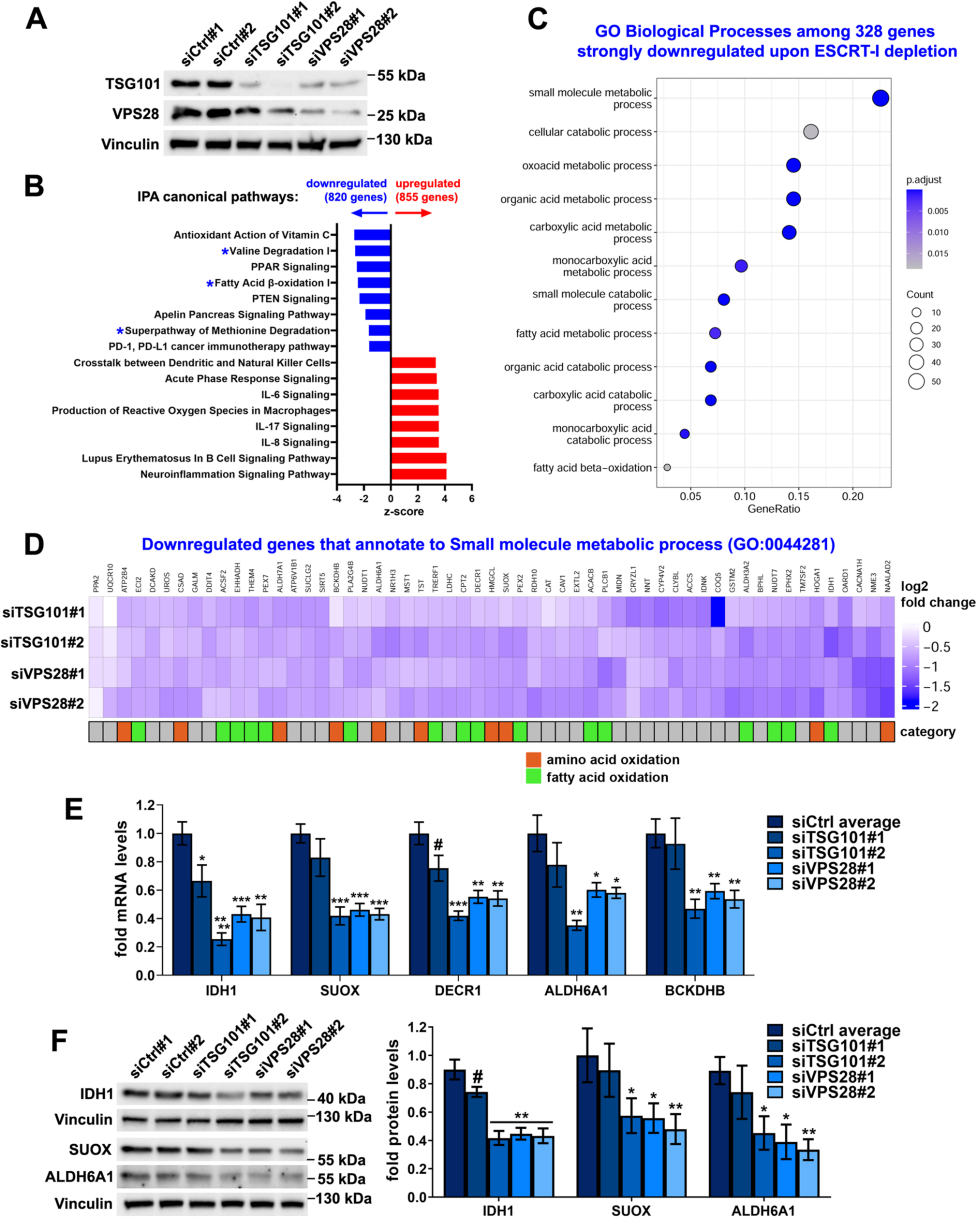
ESCRT-I dysfunction leads to reduced expression of genes involved in oxidative metabolism of amino acids and fatty acids. **A** Western blots showing the depletion efficiencies of ESCRT-I subunits, TSG101 or VPS28 using two single siRNAs for each component (siTSG101#1 or siTSG101#2, siVPS28#1 or siV2PS8#2), as compared to control cells (treated with non-targeting siRNAs, Ctrl#1 or #2) in HEK293 cells. Vinculin used as a gel loading control. **B** Ingenuity Pathway Analysis (IPA) of microarray results, showing top canonical pathways identified by annotation of genes whose expression was significantly (FDR < 0.05) downregulated or upregulated in cells depleted of ESCRT-I using two single siRNAs for each component, as compared to control cells. Blue asterisks indicate annotations related to metabolism of amino acids and fatty acids. Microarray data analysis was performed based on three independent experiments. **C** Gene ontology (GO) analysis of top biological processes identified by annotation of genes detected in microarray experiments as those with strongly downregulated expression (log2 fold change ≤ − 0.6; FDR < 0.05) upon ESCRT-I removal. **D** Heatmap visualizing expression of genes annotated to “small molecule metabolic process” (GO:0044281), whose mRNA levels were detected by microarray as downregulated after ESCRT-I removal. The genes encoding enzymes involved in oxidation of amino acids or fatty acids indicated with orange and green rectangles. **E** qPCR results showing the expression of genes encoding the indicated oxidative metabolism enzymes in cells lacking ESCRT-I, as compared to control cells, presented as fold changes with respect to averaged values measured for siCtrl#1 and #2 cells (siCtrl average). Mean values (*n* = 5 ± SEM) are presented. **F** Representative western blots (left panel), performed on the same samples as blots presented in A, showing the levels of the indicated oxidative enzymes in control or ESCRT-I-deficient cells. The graph (right panel) shows protein levels as fold change with respect to averaged values measured for control cells (siCtrl average) by densitometry analysis of western blotting bands. Vinculin was used as a gel loading control. Values derived from independent experiments and their means (*n* = 4 ± SEM) are presented. All the analyses shown in **A**–**F** were performed at three days post transfection with siRNAs (3 dpt). Statistical significance tested by comparison to siCtrl average. ^#^*P* < 0.1, *P < 0.05, **P < 0.01, ***P < 0.001, ****P < 0.0001

We have previously shown that in HEK293 cells, lack of ESCRT-I causes a very prominent activation of inflammatory NFκB-dependent signaling [30] and lysosomal starvation-related TFEB/TFE3-dependent signaling [14]. However, whether removing ESCRT-I alters the expression of metabolic genes in these cells has not been investigated. Hence, we performed a microarray analysis of HEK293 cells in which we depleted the ESCRT-I components, TSG101 or VPS28 proteins using two distinct siRNAs (designated #1 and #2) against each component (Fig. 1A) and analyzed the cells three days post transfection (3 dpt). Depletion of one of these proteins led to removal of the other one (Fig. 1A), consistent with destabilization of the whole complex that occurs upon depletion of any of its core components [31, 40]. Both siRNAs targeting VPS28 were similarly efficient in reducing the levels of VPS28 and TSG101 proteins, whereas siTSG101#1 was less efficient than siTSG101#2 in depleting TSG101 and removing VPS28 (Fig. 1A).

Among genes whose expression was commonly altered in samples with TSG101 or VPS28 depletion, we detected 855 genes with significantly upregulated expression and 820 with significantly downregulated expression, as compared to control cells (Fig. 1B). These genes were functionally annotated using the Ingenuity Pathway Analysis. As expected from our previous studies [14, 29, 30], ESCRT-I depletion led to elevated expression of many genes involved in inflammation and stress response (Fig. 1B).

Importantly, we observed that among genes with reduced expression in cells lacking ESCRT-I were those annotated to oxidative breakdown of amino acids and fatty acids (Fig. 1B). Our further analysis using Gene Ontology Biological Processes database, focusing only on 328 genes with the strongest downregulation (log2 fold change ≤ − 0, 6), showed that ESCRT-I depletion led to reduced expression of over 50 genes involved in “small molecule metabolism” (Fig. 1C–D). This list included mainly genes encoding enzymes responsible for oxidative metabolism of carboxylic acid-containing molecules such as fatty acids and amino acids (Fig. 1D**, **Table S1). Given that these genes encode enzymes involved in oxidation of such molecules, we refer to them hereafter as “amino acid or fatty acid oxidation genes”.

To validate the transcriptomic results, we focused on genes encoding IDH1 and DECR1 enzymes, that participate in fatty acid metabolism [41, 42], as well as SUOX, ALDH6A1 and BCKDHB [43–45] that are involved in amino acid metabolism. By quantitative RT-PCR (qRT-PCR), we independently confirmed the reduced mRNA levels of each of these enzymes in HEK293 cells lacking TSG101 or VPS28 (Fig. 1E). However, siTSG101#1, that was less efficient in depleting TSG101 and VPS28 proteins than siTSG101#2 (shown in Fig. 1A), had a weaker effect on the mRNA levels of the analyzed enzymes. This suggested that high efficiency of TSG101 depletion has to be achieved to lower the expression of amino acid or fatty acid oxidation genes. To investigate whether reduced expression of the chosen genes occurred also in other cell types, we performed qRT-PCR analysis in HepG2 hepatoblastoma cell line with siRNA-mediated removal of TSG101 or VPS28 proteins using siTSG101#2 and siVPS28#1 (Fig. S1A) or CRISPR-Cas9-mediated knock-out of *TSG101* gene (Fig. S1B–C). The efficiencies of using siRNAs or CRISPR-Cas9 system in these cells were assessed by qRT-PCR (Fig. S1A) or western blotting (Fig. S1B), respectively. In each case, deficiency of ESCRT-I complex led to reduced expression of analyzed amino acid or fatty acid oxidation genes (Fig. S1A and C).

To address whether changes in the expression of amino acid or fatty acid oxidation genes observed upon ESCRT-I deficiency could affect cell metabolism, we tested protein levels of chosen enzymes in HEK293 cells by western blotting. We confirmed that siRNA-mediated depletion of TSG101, using siTSG101#1, or VPS28, using siVPS28#1 or #2, at 3 dpt caused reduced abundance of IDH1, SUOX and ALDH6A1 oxidative enzymes (Fig. 1F). Thus, the transcriptomic analysis and its validation demonstrated that ESCRT-I deficiency causes reduced gene expression and protein levels of enzymes involved in oxidation of small molecules such as amino acids and fatty acids.

### ESCRT-I deficiency causes intracellular accumulation of lipids, including phospholipids that accumulate in the enlarged ER

**Fig. 2:**
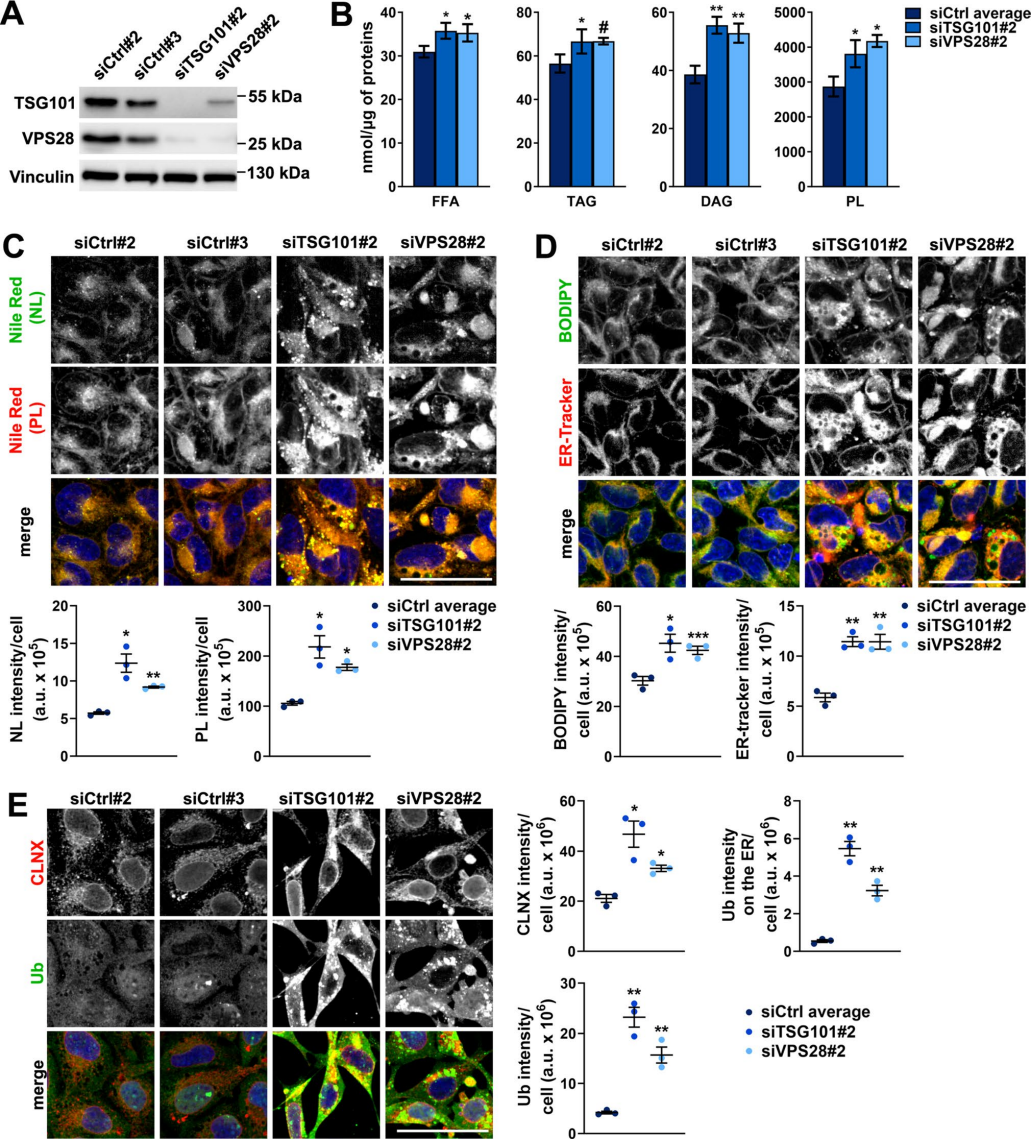
Cells lacking ESCRT-I have elevated levels of lipids and enlarged ER. **A** Western blots showing the efficiencies of siRNA-mediated depletions of ESCRT-I subunits, TSG101 or VPS28 (cells treated with siTSG101#2 or siV2PS8#2), as compared to control conditions (two non-targeting siRNAs, Ctrl#2 or #3) in HEK293 cells. Vinculin used as a gel loading control. **B** Results of gas chromatography followed by mass spectrometry (GC–MS) showing intracellular levels of free fatty acids—FFA, triglycerides—TG, diacylglycerides—DAG or phospholipids—PL (shown as nmol/µg of proteins) in control or ESCRT-I-depleted cells. Values derived from independent experiments and their means (*n* = 4 ± SEM) are presented. Values for siCtrl average are averaged values measured for cells transfected with siCtrl#2 or siCtrl#3. **C**–**D** Maximum intensity projection confocal images of live control or ESCRT-I-depleted cells. The images show the intracellular distribution of neutral lipids—NL (green), or phospholipids—PL (red) stained with Nile Red dye (shown in C) as well as the intracellular distribution of NLs stained with BODIPY 493/503 and the ER stained with ER-tracker (green or red, respectively in **D**). The dot plots show total fluorescence intensities per cell (expressed in arbitrary units, a.u.), as compared to averaged values measured for cells transfected with siCtrl#2 or siCtrl#3 (siCtrl average). Average number of cells analyzed per condition was 2936 for siCtrl#2, 2768 for siCtrl#3, 1419 for siTSG101#2 and 1871 for siVPS28#2. **E** Maximum intensity projection confocal images of fixed control or ESCRT-I-depleted cells stained using antibodies recognizing CLNX protein (red) or mono- and polyubiquitinated protein conjugates (Ub; green). The dot plots show total fluorescence intensities per cell (expressed in arbitrary units, a.u.), as compared to averaged values measured for cells transfected with siCtrl#2 or siCtrl#3 (siCtrl average). Average number of cells analyzed per condition was 4414 for siCtrl#2, 4481 for siCtrl#3, 2126 for siTSG101#2 and 2473 for siVPS28#2. Cell nuclei in **C**–**E** marked with Hoechst stain (blue). Scale bars, 50 μm. Dot plot values in **C**–**E** derived from independent experiments (dots) and their means (*n* = 3 ± SEM) are presented. All the analyses shown in **B**–**E** were performed at three days post transfection with siRNAs (3 dpt). Statistical significance tested by comparison to the siCtrl average values. ^#^*P* < 0.1, *P < 0.05, **P < 0.01, ***P < 0.001

Reduced expression of genes encoding enzymes of fatty acid oxidation is often associated with intracellular accumulation of lipids [46]. Moreover, we observed increased expression of several genes encoding enzymes of biosynthesis of various types of lipids (fatty acids, triglycerides or phospholipids) in cells lacking ESCRT-I (Fig. S2A). Thus, we investigated the abundance of such lipids in cells with TSG101 or VPS28 depletion using single siRNAs (siTSG101#2 and siVPS28#2) against each protein (Fig. 2A).

To assess the intracellular content of fatty acids, we performed gas chromatography followed by mass spectrometry (GC–MS) (Fig. 2B). We measured free fatty acids (FFAs) and fatty acid-containing lipids, i.e., triglycerides (TGs), diacylglycerides (DAGs) and phospholipids (PLs). Depletion of TSG101 or VPS28 led to slightly elevated levels of FFAs and TGs and to an even stronger increase in membrane lipids, DAGs and PLs (Fig. 2B). To verify elevated lipid levels, we stained control and ESCRT-I-depleted cells with Nile Red dye (NR) and imaged them by confocal microscopy. NR emits green fluorescence, when bound to neutral lipids (NLs, that include cholesterol or TGs), or red fluorescence, when bound to PLs [47]. Consistent with the GC–MS results, we observed that lack of ESCRT-I led to increased levels of both NLs and PLs (Fig. 2C). We confirmed elevated abundance of NLs in cells lacking TSG101 or VPS28 by using BODIPY 493/503 dye (Fig. 2D) that emits green fluorescence when bound to these lipids [48]. The amounts of NLs were increased primarily in lipid droplets—LDs (Fig. 2C–D), identified as punctate structures positive for both NLs and PLs [49]. The LDs were more abundant upon ESCRT-I depletion (Fig. S2B), but had similar mean size as in control cells (Fig. S2C). PLs accumulated in ESCRT-I-deficient cells also outside of LDs, in a region resembling the ER by shape and localization (Fig. 2C). As PLs are the most abundant lipids in the cell, that build cellular membranes, we reasoned that their accumulation in the ER region could reflect an increase of ER volume. Indeed, we noticed that cells lacking TSG101 or VPS28 had a strongly increased ER content as measured by confocal microscopy using ER-tracker Red dye (Fig. 2D) and antibodies recognizing calnexin (CLNX), an ER-resident protein (Fig. 2E).

In mammalian cells the ER size is restricted by its selective autophagic degradation that occurs in an ubiquitin-dependent manner [50]. To address whether the enlargement of the ER due to lack of ESCRT-I could be a consequence of an impaired ER degradation, we quantified the amount of ubiquitin on the CLNX-positive compartment. Similarly as we reported for RKO colon cancer cells [14], depletion of TSG101 or VPS28 in HEK293 cells led to a strong intracellular accumulation of ubiquitin (Fig. 2E), likely due to the impairment of multiple ESCRT-I-mediated degradation processes [14]. Some of the accumulated ubiquitin was enriched in the CLNX-positive compartment (Fig. 2E) indicating that ESCRT-I deficiency may impair the ER-phagy.

Hence, we discovered that lack of TSG101 or VPS28 leads to increased abundance of FFAs and lipids, such as PLs that accumulate in intracellular membranes including the enlarged ER. Although increased amount of fatty acid-containing lipids in cells lacking ESCRT-I could be in part due to their impaired lysosomal degradation, for instance ER-phagy inhibition, it could also result from reduced oxidative breakdown of FFAs and increased lipid biosynthesis, as implied by the changes in gene expression described above (Fig. 1 and S2).

### ESCRT-I deficiency does not impair mitochondrial biogenesis or ATP-linked mitochondrial respiration

**Fig. 3:**
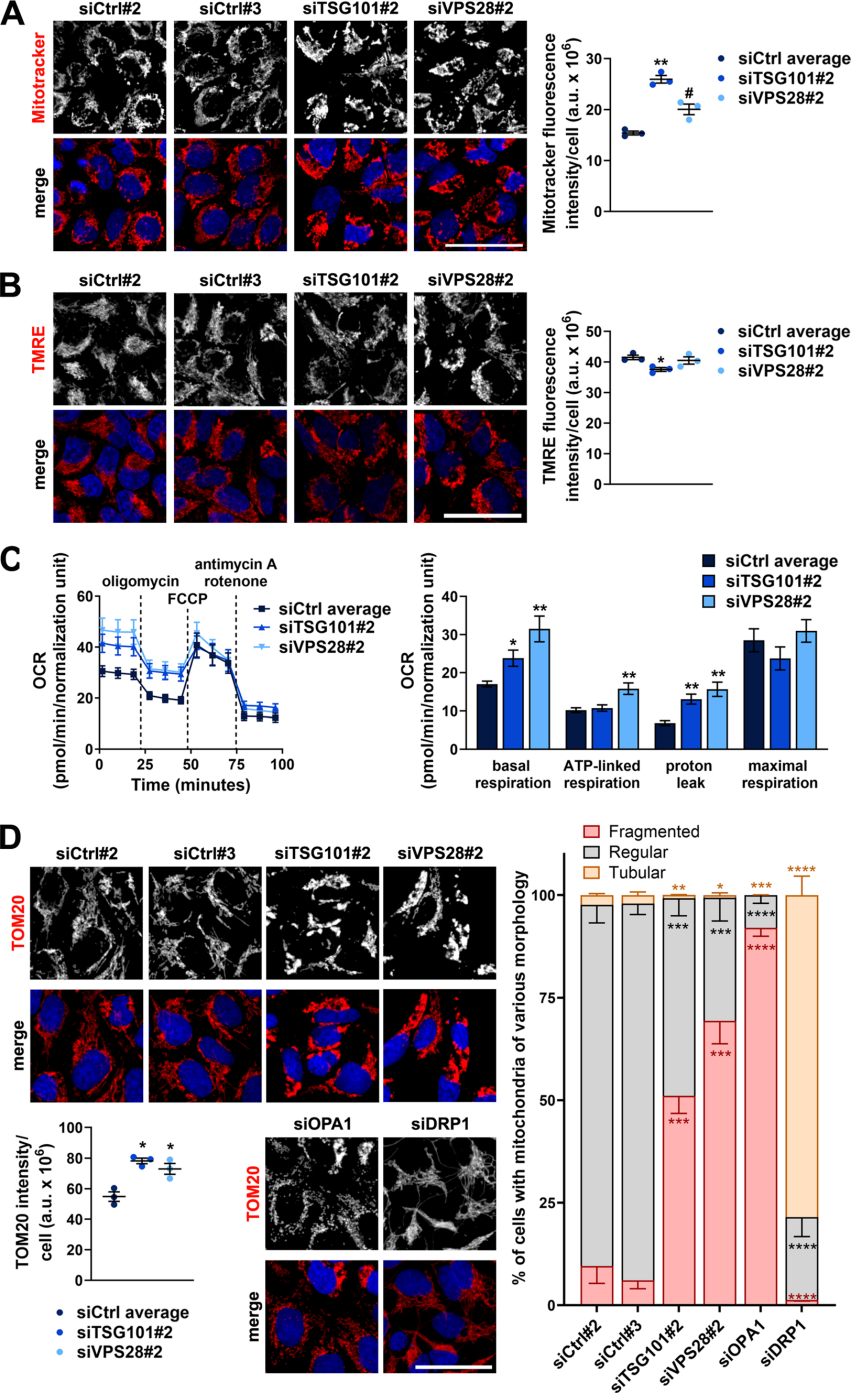
The abundance of functional mitochondria and the rate of ATP-linked mitochondrial respiration are not reduced upon ESCRT-I deficiency. **A**–**B** Maximum intensity projection confocal images of live control (treated with non-targeting siRNAs, Ctrl#2 or #3) or ESCRT-I-depleted HEK293 cells (treated with siTSG101#2 or siV2PS8#2). The images show the intracellular content and distribution of all mitochondria stained with MitoTracker Deep Red FM dye (red in A) and of mitochondria with proper membrane potential stained with Tetramethylrhodamine, Ethyl Ester (TMRE, red in B). Cell nuclei marked with Hoechst 33342 stain (blue). Scale bar, 50 μm. The dot plots show total fluorescence intensities per cell (expressed in arbitrary units, a.u.), as compared to averaged values measured for cells transfected with siCtrl#2 or siCtrl#3 (siCtrl average). Values derived from independent experiments (dots) and their means (*n* = 3 ± SEM) are presented. Average number of cells analyzed per condition was 2542 for siCtrl#2, 2527 for siCtrl#3, 1464 for siTSG101#2 and 1729 for siVPS28#2. **C** A representative time-series graph (left) showing changes of oxygen consumption rate (OCR) with time (pmol/min) in control or ESCRT-I-depleted cells upon: basal respiration (untreated cells; time-points 1–3), inhibition of ATP-linked respiration (oligomycin treatment; time-points 4–6), maximal respiration (FCCP treatment; time-points 7–9) and non-mitochondrial respiration (antimycin A and rotenone treatment; time-points 10–12). The bar graph (right) shows the intensity of the indicated processes calculated based on the results shown in the time-series graph. The results shown in both graphs were normalized to cell number reflected by DNA staining with Hoechst 33342 dye. Mean values in both graphs derived from technical repetitions of one experiment (*n* = 4 or 5 ± SEM) are presented. **D** Maximum intensity projection confocal images of fixed control, ESCRT-I-depleted, OPA1-depleted or DRP1-depleted cells stained using antibodies recognizing TOM20 protein (red). Cell nuclei marked with DAPI stain (blue). Scale bar, 50 μm. The dot plot (bottom left) shows total fluorescence intensity per cell (expressed in arbitrary units, a.u.), as compared to averaged values measured for cells transfected with siCtrl#2 or siCtrl#3 (siCtrl average). Values derived from independent experiments (dots) and their means (*n* = 3 ± SEM) are presented. Graph on the right shows percentage of cells with fragmented, regular or tubular mitochondria. Average number of cells analyzed per condition was 1304 for siCtrl#2, 1132 for siCtrl#3, 598 for siTSG101#2, 765 for siVPS28#2, 1170 for siDRP1 and 896 for siOPA1. All the analyses shown in A-D were performed at three days post transfection with siRNAs (3 dpt). Statistical significance tested by comparison to the siCtrl average values. ^#^*P* < 0.1, *P < 0.05, **P < 0.01, ***P<0.001, ****P<0.0001

By GO Cellular Component analysis of genes with strongly reduced expression in cells lacking TSG101 or VPS28, we identified many genes that encode mitochondrial proteins (Fig. S3A, Table S2; around 50 genes that included many of the amino acid or fatty acid oxidation genes indicated in Fig. 1D). Reduced expression of genes encoding mitochondrial proteins could potentially affect mitochondrial biogenesis. However, we did not observe a general reduction in the expression of genes encoding structural mitochondrial proteins or core components of the citric acid cycle or oxidative phosphorylation. To investigate the consequence of ESCRT-I deficiency on the abundance of mitochondria, we performed confocal microscopy analysis of HEK293 cells stained with MitoTracker Deep Red FM dye. We observed that MitoTracker staining was not reduced in the absence of ESCRT-I at 3 dpt, on the contrary, it was increased (Fig. 3A). These data indicated that ESCRT-I deficiency did not impair the biogenesis of mitochondria.

As ESCRTs are required for mitochondria degradation in lysosomes [26, 27], the accumulation of mitochondria observed in cells lacking TSG101 or VPS28 could be a result of impaired autophagic removal of damaged mitochondria. Hence, to address whether ESCRT-I deficiency affects the functionality of mitochondria, we measured mitochondrial membrane potential using Tetramethylrhodamine Ethyl Ester (TMRE) dye that stains properly functioning mitochondria. By confocal microscopy, we observed that the TMRE fluorescence intensity was slightly reduced upon TSG101 depletion and not altered upon VPS28 depletion (Fig. 3B). Hence, the overall mitochondrial membrane potential did not increase upon ESCRT-I deficiency (Fig. 3B) as it was the case for overall mitochondria content (shown in Fig. 3A). This analysis suggested that cells lacking ESCRT-I retain functional mitochondria but also accumulate excess mitochondria that do not have proper membrane potential.

To address whether the reduced expression of amino acid or fatty acid oxidation genes or the accumulation of damaged mitochondria upon ESCRT-I deficiency affect mitochondrial respiration, we measured oxygen consumption rate (OCR) using the Agilent Seahorse XFe24 Analyzer. Control HEK293 cells showed typical OCR curve, indicating basal respiration (as a sum of ATP-linked and proton leak-related processes) that was lower than maximal respiratory capacity (Fig. 3C). However, cells lacking ESCRT-I components had elevated basal respiration, reaching maximal capacity, due to increased proton leak (Fig. 3C). The elevated proton leak was consistent with the above-described accumulation of damaged mitochondria upon ESCRT-I deficiency (shown in Fig. 3A–B). Importantly, the OCR linked to ATP production was not impaired in cells lacking ESCRT-I depletion (Fig. 3C). It was not affected by depletion of TSG101 and was even elevated upon VPS28 depletion as compared to control cells (Fig. 3C).

Overall, ESCRT-I deficiency does not affect the abundance of mitochondria with proper membrane potential. Moreover, despite strongly impaired lysosomal degradation of proteins [14, 30] and lipids (shown in Fig. 2B–C), as well as reduced levels of enzymes involved in amino acid and fatty acid degradation (shown in Fig. 1F), ESCRT-I depletion does not impair ATP-dependent mitochondrial respiration. Hence, the observed metabolic changes in cells lacking ESCRT-I are not due to general mitochondria malfunction.

### Lack of ESCRT-I promotes fragmentation of mitochondria

Analyzing the confocal microscopy images after MitoTracker staining (shown in Fig. 3A), we noticed that cells lacking TSG101 or VPS28 may have altered morphology of mitochondria. To address this in detail, we analyzed by confocal microscopy the intracellular distribution of a mitochondrial marker, TOM20 protein in HEK293 cells. Consistent with higher mitochondrial abundance in ESCRT-I-deficient cells, that we observed using MitoTracker, these cells had elevated staining intensity of TOM20 as compared to control cells (Fig. 3D). Of note, the quantitative analysis of the confocal images pointed out that in part accumulation of ubiquitin in cells lacking ESCRT-I occurs on the TOM20-positive compartment (Fig. S3B). However, as we did not observe clear colocalization of TOM20 with ubiquitin accumulated in cells lacking ESCRT-I (Fig. S3B), we could not conclude that the elevated signal intensity of MitoTracker (shown in Fig. 3A) or TOM20 (Fig. 3D) in cells lacking ESCRT-I is due to accumulation of non-degraded, ubiquitinated mitochondria.

Mitochondrial morphology is determined by fusion and fission processes mediated by a number of regulators including dynamin-related protein 1 (DRP1), the master regulator of mitochondrial fission, and optic atrophy 1 (OPA1) protein, that facilitates mitochondrial fusion [51]. In order to assess how lack of ESCRT-I affects the morphology of mitochondria, we used the supervised machine learning module of the software dedicated for the Opera high-content screening microscope, with which we obtained the confocal images. This allowed us to compare the distribution of TOM20 protein in cells lacking TSG101 or VPS28 to TOM20 distribution in cells lacking DRP1 or OPA1, based on a large number of confocal images. According to this analysis, most of control HEK293 cells had regularly shaped mitochondria with only around 10% of cells with fragmented mitochondria identified (Fig. 3D). As expected, siRNA-mediated depletion of OPA1 caused mitochondrial fragmentation (over 90% of cells), whereas most of the cells with depletion of DRP1 had tubular mitochondria and barely any cells lacking DRP1 (around 1%) had fragmented mitochondria (Fig. 3D). Interestingly, ESCRT-I deficiency caused an increased percentage of cells (50%-70%) identified as those containing fragmented mitochondria (Fig. 3D).

These data indicated that the presence of functional ESCRT-I promotes mitochondria fusion or inhibits mitochondria fission. The molecular mechanisms underlying this regulation are yet to be discovered.

### Cells lacking ESCRT-I activate glycolytic metabolism

**Fig. 4:**
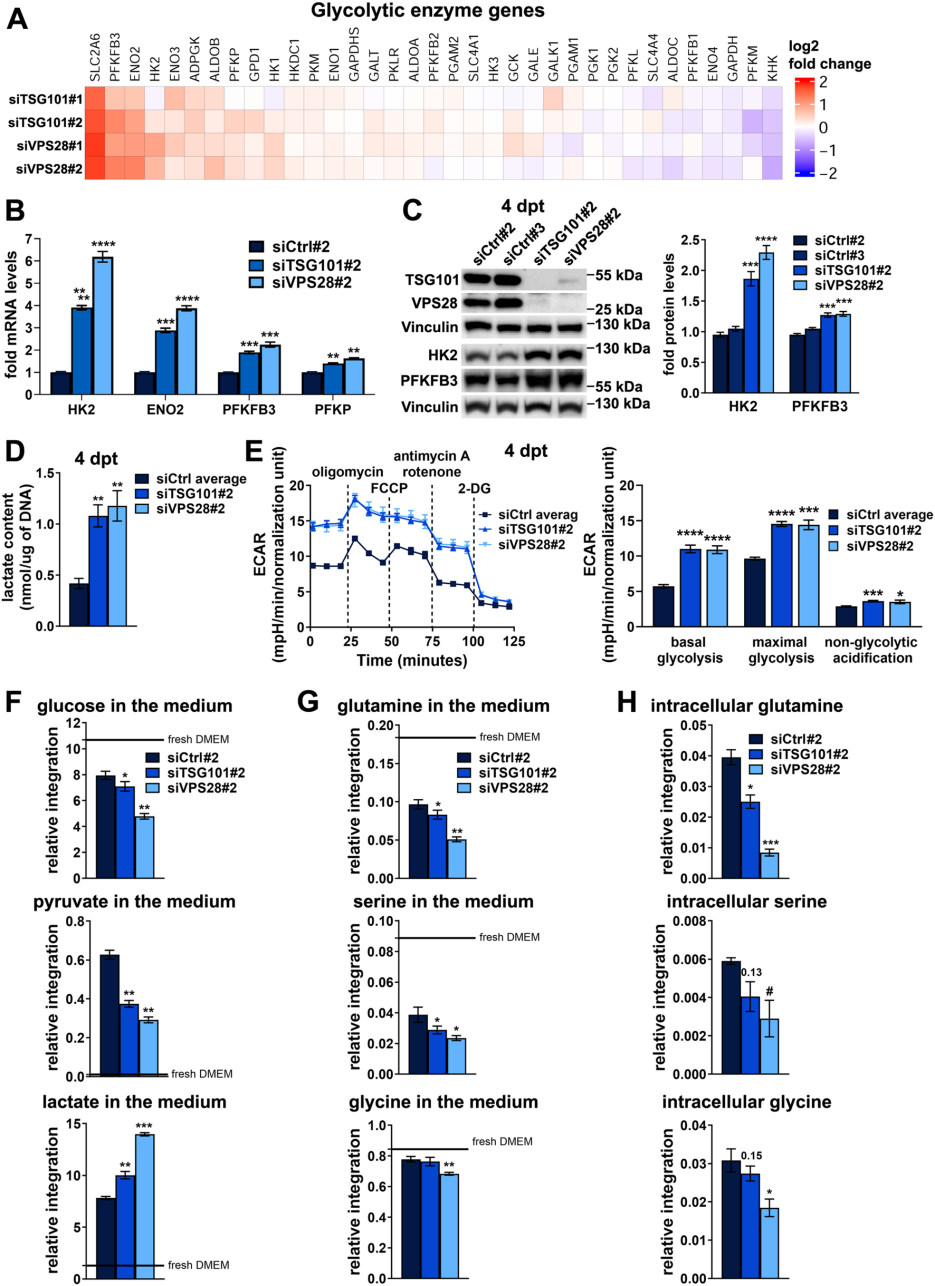
ESCRT-I dysfunction leads to increased aerobic glycolysis and other metabolic changes reminiscent of the Warburg effect. **A** Heatmap visualizing microarray results regarding the expression of genes encoding enzymes involved in glycolysis in HEK293 cells after removal of ESCRT-I using two siRNAs for each component (siTSG101#1 or siTSG101#2, siVPS28#1 or siVPS28#2), as compared to control cells (treated with non-targeting siRNAs, Ctrl#1 or #2). Microarray data analysis was performed based on three independent experiments at three days post transfection with siRNAs (3 dpt). **B** qPCR results showing the expression of genes encoding glycolytic enzymes in cells depleted of TSG101 or VPS28 using single siRNAs (siTSG101#2 or VPS28#2), as compared to control cells (treated with non-targeting siRNA, Ctrl#2) at 4 dpt. The mean values (*n* = 4 ± SEM) presented as fold changes with respect to values for control cells. **C** Representative western blots (left panel) showing the levels of TSG101, VPS28 and the indicated glycolytic enzymes in control (treated with non-targeting siRNAs, Ctrl#2 or #3) or ESCRT-I-depleted (siTSG101#2 or siVPS28#2) cells. The graph (right panel) shows protein levels as fold change with respect to averaged values measured for siCtrl#2 and #3 assessed by densitometry analysis of western blotting bands. Vinculin was used as a gel loading control. Mean values (*n* = 4 ± SEM) are presented. **D** Intracellular levels of lactate (shown as nmol/µg of DNA) in control (siCtrl#1 or #2) or ESCRT-I-deficient cells. Mean values (*n* = 3 ± SEM) are presented. Values for siCtrl average are averaged values measured for cells transfected with siCtrl#1 or siCtrl#2. **E** A representative time-series graph (left) showing changes of extracellular acidification rate (ECAR) with time (mpH/min) in control (siCtrl#2 or #3) or ESCRT-I-depleted cells at 4 dpt upon: basal respiration (untreated cells; time-points 1–3), inhibition of ATP-linked respiration (oligomycin treatment; time-points 4–6), maximal respiration (FCCP treatment; time-points 7–9), non-mitochondrial respiration (antimycin A and rotenone treatment; time-points 10–12) and inhibition of glycolysis (2-DG treatment; time-points 13–15). The bar graph (right) shows the intensity of the indicated processes calculated based on the results shown in the time-series graph. The results shown in both graphs were normalized to cell number reflected by DNA staining with Hoechst 33342 dye. Mean values in both graphs derived from technical repetitions of one experiment (*n* = 5 ± SEM) are presented. **F**–**G** Abundance of chosen metabolites detected by ^1^H-NMR analysis in the medium from control or ESCRT-I-deficient cells as compared to levels in fresh DMEM (*n* = 4 ± SEM). The medium was collected after 24 h of cell culture from 3 to 4 dpt. **H** Intracellular levels of chosen amino acids detected by ^1^H-NMR analysis of metabolites in pellets of control or ESCRT-I-deficient cells at 4 dpt (*n* = 4 ± SEM). Statistical significance in **B**, **C**, **F**, **G** and **H **tested by comparison to siCtrl#2, whereas in **D** and **E** tested by comparison to siCtrl average. ^#^*P* < 0.1, *P < 0.05, **P < 0.01, ***P < 0.001, ****P < 0.0001

Small mitochondria are characteristic for cells that base their metabolism on aerobic glycolysis [52, 53]. Hence, we reasoned that the reduced expression of amino acid or fatty acid oxidation genes in ESCRT-I-deficient cells could be associated with changes in expression of genes involved in glucose catabolism. Although the Ingenuity Pathway Analysis of genes with commonly induced expression upon TSG101 or VPS28 depletion (Fig. 1B) did not indicate such annotation, we investigated in more detail the transcriptomic results focusing on genes encoding enzymes involved in glycolysis, hence metabolism of glucose to pyruvate (Fig. 4A), and in conversion of pyruvate to acetyl-CoA (Fig. S4A). We found that ESCRT-I deficiency caused elevated expression of several genes that encode glycolytic enzymes (Fig. 4A) but had no particular effect on the expression of genes responsible for pyruvate to acetyl-CoA conversion (Fig. S4A). By qRT-PCR analysis performed at 3 dpt (the same time-point as of the transcriptomic analysis), we verified the increased expression of HK2, ENO2, PFKFB3 and PFKP glycolytic enzymes in HEK293 cells upon depletion of VPS28 with two independent siRNAs (Fig. S4B). However, such increase was not observed in cells transfected with siTSG101#1 and occurred only for ENO2 and PFKFB3 in cells transfected with siTSG101#2 (Fig. S4B).

Reasoning that 3 dpt could be too early to observe a full effect on glycolytic gene expression in cells lacking TSG101, we performed the analysis in cells transfected with siTSG101#2 or siVPS28#2 at 4 dpt and observed a prominent increase in the expression of all analyzed glycolytic genes (Fig. 4B). As verified by western blotting, depletion of TSG101 or VPS28 at 4 dpt was very efficient (Fig. 4C). The upregulated expression of genes encoding ENO2, PFKFB3 and PFKP (but not HK2) also occurred in ESCRT-I-deficient HepG2 cells at 3 dpt (Fig. S4C–D). As in HEK293 cells, we observed a stronger increase in the expression of these glycolytic genes in HepG2 cells with siRNA-mediated VPS28 depletion than upon TSG101 depletion (Fig. S4C). However, CRISPR-Cas9-mediated TSG101 depletion led to clearly upregulated expression of genes encoding ENO2, PFKFB3 and PFKP (Fig. S4C).

Next, we addressed whether increased expression of genes encoding particular glycolytic enzymes translates into their higher abundance and increased glycolytic metabolism. Analyzing the cells at 4 dpt, we observed elevated protein levels of HK2 and PFKFB3 enzymes upon depletion of both VPS28 or TSG101 (Fig. 4C). To address whether ESCRT-I-deficient HEK293 cells activate glycolytic metabolism, we measured the intracellular content of lactate, the product of anaerobic glucose metabolism. We found that at 4 dpt, cells lacking TSG101 or VPS28 had clearly elevated intracellular levels of lactate (Fig. 4D). To verify the effect of ESCRT-I deficiency on aerobic glycolysis, we used the Agilent Seahorse XFe24 Analyzer to measure the extracellular acidification rate (ECAR) that is increased upon release of lactate [54]. This analysis showed that, as compared to control cells, cells lacking TSG101 or VPS28 had elevated ECAR, primarily due to glycolysis (Fig. 4E), although their non-glycolytic acidification was also slightly elevated (Fig. 4E). Of note, depletion of ESCRT-I components for 4 days caused an increase of both, basal glycolysis as well as maximal glycolytic capacity (Fig. 4E).

Hence, in cells lacking ESCRT-I, the inhibited expression of amino acid or fatty acid oxidation genes is associated with activated expression of glycolytic genes and induction of glycolytic metabolism. These results suggest that functional ESCRT-I may promote oxidative metabolism of lysosome-derived nutrients over glycolytic metabolism.

### Metabolic reprogramming upon ESCRT-I deficiency resembles the Warburg effect

Collectively, the above-described results suggested that lack of ESCRT-I may lead to metabolic reprogramming similar to the Warburg effect that is characterized by increased consumption of glucose and some amino acids [10]. To verify this, we investigated the effect of ESCRT-I deficiency on consumption of metabolites using nuclear magnetic resonance (^1^H-NMR) approach. We analyzed the changes in abundance of metabolites in the medium of cells during 24 h of culture, from 3 to 4 dpt, as well as assessed the intracellular metabolite levels at 4 dpt (Tables S3–S6). ^1^H-NMR allowed to detect various extracellular and intracellular metabolites including most of the amino acids (Fig. S5A-B, Tables S3 and S4). However, glucose and pyruvate were detected only in the medium but not inside the cells (Fig. S5A–B, Tables S3 and S4). We observed that control cells consumed some glucose from the medium but also released pyruvate and lactate (Fig. 4F). Importantly, cells lacking TSG101 or VPS28 had increased glucose consumption, lower pyruvate release and higher lactate release (Fig. 4F), indicating elevated conversion of medium-derived glucose through pyruvate into lactate.

Upon the Warburg effect, elevated glucose consumption in cells is associated with increased uptake of glutamine, serine and glycine. We observed that depletion of TSG101 or VPS28 led to increased usage of glutamine and serine but not glycine from the medium (Fig. 4G). Moreover, cells lacking ESCRT-I had reduced intracellular abundance of glutamine, glycine and (less significantly) serine (Fig. 4H). Lower medium and/or intracellular levels of these amino acids suggested their increased utilization. Of note, ESCRT-I deficiency had no effect on extracellular or intracellular levels of BCAAs (leucine, isoleucine and valine) (Fig. S5C–D), showing that elevated glutamine, serine and glycine consumption was not due to general alterations in amino acid metabolism.

The increased glutamine consumption occurs in cells with the Warburg effect, among other purposes, to maintain ATP production, that would otherwise be impaired due to shift to aerobic glycolysis [5]. Accordingly, our ^1^H-NMR analysis showed that lack of TSG101 or VPS28 did not affect the intracellular ATP or ADP levels, as compared to control cells (Fig. S5E). This was consistent with the OCR analysis which showed that ESCRT-I deficiency did not impair ATP-linked oxidation (shown in Fig. 3C). Among molecules biosynthesized from one-carbon metabolism of glycine and serine, cells exerting the Warburg effect produce high amounts of glutathione [55]. Consistently, we observed elevated glutathione abundance in cells lacking TSG101 or VPS28 (Fig. S5F).

Hence, we confirmed that ESCRT-I dysfunction leads to metabolic reprogramming resembling the Warburg effect. Noteworthy, this reprogramming is more pronounced in cells lacking VPS28 than in cells lacking TSG101 (Fig. 4F–H), potentially due to faster response in upregulation of glycolytic gene expression upon VPS28 depletion (shown in Fig**.** S4B and 4B).

### mTORC1 signaling is not implicated in regulation of cell metabolism upon ESCRT-I depletion

Next, we sought to investigate which signaling pathways could be involved in the altered expression of metabolic genes upon ESCRT-I depletion. Cell metabolism is largely controlled by mTORC1 signaling that regulates amino acid, fatty acid and glucose metabolism. We have previously found that in RKO colorectal cancer cells, ESCRT-I deficiency does not affect general mTORC1 signaling, for instance phosphorylation of mTOR kinase target S6K1 [14]. To verify whether general mTORC1 signaling is affected in HEK293 cells lacking ESCRT-I, we assessed the phosphorylation of S6K1 kinase target, S6 protein. We observed that S6 phosphorylation is a good indicator of mTORC1 activity as treatment with INK128 compound (mTOR kinase inhibitor) caused a very strong reduction of this phosphorylation levels in control HEK293 cells (Fig. S6A). Importantly, ESCRT-I deficiency did not affect basal S6 phosphorylation and treating TSG101- or VPS28-depleted cells with INK128 reduced S6 phosphorylation to the same level as observed in INK128-treated control cells (Fig. S6A). Hence, we confirmed that in HEK293 cells, similarly to RKO cells, lack of ESCRT-I has no effect on general mTORC1 signaling.

As we have reported previously, HEK293 cells show a strong induction of TFEB/TFE3 signaling upon ESCRT-I depletion, that is a hallmark of activating the substrate-specific mTORC1 signaling due to lysosomal starvation [14]. Intriguingly, TFEB transcription factor was shown to stimulate the expression of genes involved in fatty acid oxidation [38]. Hence, to investigate any potential impact of TFEB/TFE3 signaling on regulating the transcription of amino acid or fatty acid oxidation genes upon ESCRT-I depletion, we depleted simultaneously TFEB and TFE3 using siRNA and analyzed the cells at 3 dpt. As tested by qRT-PCR, depletion efficiencies of TSG101 and VPS28 were equally good upon single depletion or co-depletion with TFEB/TFE3 (Fig. S6B) and depletion efficiencies of TFEB and TFE3 were equally good upon their double depletion or triple depletion with TSG101 or VPS28 (Fig. S6C). We observed that TFEB/TFE3 depletion alone led to significantly reduced mRNA levels of ALDH6A1 and BCKDHB but not of other analyzed enzymes in control cells (Fig. S6D). However, co-depletion of TFEB/TFE3 and ESCRT-I potentiated the reduced expression of all analyzed metabolic genes that occurs due to removal of TSG101 or VPS28 (Fig. S6D). Hence, we found that although TFEB/TFE3 signaling may promote the expression of genes involved in oxidative metabolism of amino acids and fatty acids, ESCRT-I depletion reduces expression of these genes despite TFEB/TFE3 signaling activation.

Collectively, we did not find evidence that the transcriptional regulation of cell metabolism observed in cells lacking ESCRT-I occurred due to the regulation of general or substrate-specific mTORC1 signaling.

### Activation of canonical NFκB signaling contributes to the reduced expression of amino acid or fatty acid oxidation genes

**Fig. 5:**
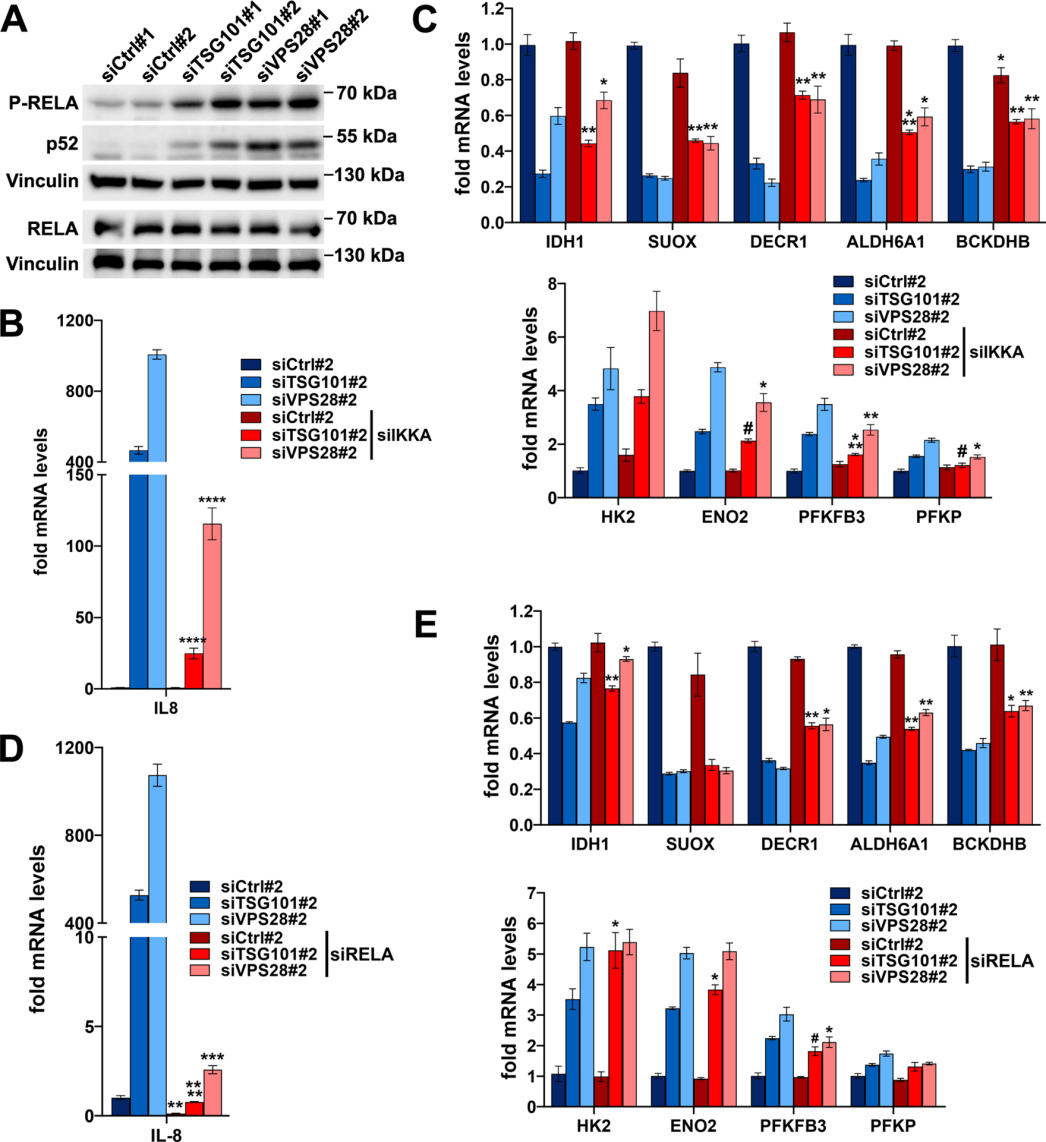
The reduced expression of genes encoding enzymes of amino acid or fatty acid oxidation in cells lacking ESCRT-I occurs in part due to activation of NFκB signaling. **A** Western blots, performed on the same samples as blots presented in Fig. 1A, showing the levels of phosphorylated (P-RELA) and total RELA protein as well as p52 protein in HEK293 cells depleted of ESCRT-I using two single siRNAs for each component (siTSG101#1 or siTSG101#2, siVPS28#1 or siVPS28#2), as compared to control cells (treated with non-targeting siRNAs, Ctrl#1 or #2). The analysis was performed at three days post siRNA transfection (3 dpt) with vinculin used as a gel loading control. **B**–**C** qPCR results showing the expression of genes encoding IL-8 (in B) or indicated oxidative (top graph in C) or glycolytic enzymes (bottom graph in C) in control (siCtrl#2) or ESCRT-I-deficient (siTSG101#2 or siVPS28#2) cells with single depletion or with co-depletion of IKKA (siIKKA) at 4 dpt. The results presented as fold changes with respect to siCtrl#2. Mean values (*n* = 4 ± SEM) are presented. **D**–**E** qPCR results showing the expression of genes encoding IL-8 (in D) or indicated oxidative (top graph in E) or glycolytic enzymes (bottom graph in E) in control (siCtrl#2) or ESCRT-I-deficient (siTSG101#2 or siVPS28#2) cells with single depletion or with co-depletion of RELA (siRELA) at 4 dpt, presented as fold changes with respect to siCtrl#2. Mean values (*n* = 3 ± SEM) are presented. Statistical significance in (**B**–**E**) tested by comparison of cells lacking only TSG101 or VPS28 to those lacking these proteins and IKKA or RELA. ^#^*P* < 0.1, *P < 0.05, **P < 0.01, ***P < 0.001, ****P < 0.0001

Having ruled out any clear contribution of mTORC1 signaling to regulation of metabolic gene expression upon ESCRT-I deficiency, we addressed a potential involvement of inflammatory/stress response pathways. Consistent with our previous study [30], ESCRT-I depletion in HEK293 cells led to the induction of canonical and non-canonical NFκB signaling, assessed by the activation of two relevant NFκB transcription factors, namely: elevated phosphorylation of RELA protein and increased levels of p52 protein (Fig. 5A), respectively [30, 31]. The two NFκB pathways require activation of distinct upstream kinase complexes that however share a common component, Inhibitory-κB Kinase α (IKKA) [56]. Given that the canonical, RELA-dependent, NFκB pathway may repress the expression of oxidative metabolism genes and promote glycolysis [34, 35, 57], we tested whether activation of NFκB signaling was responsible for the changes in metabolic gene expression observed upon ESCRT-I deficiency.

First, we simultaneously depleted TSG101 or VPS28 proteins with IKKA kinase to block the activation of both, the canonical and non-canonical NFκB branches upon ESCRT-I deficiency and analyzed the cells at 4 dpt. As observed by qRT-PCR analysis, we achieved efficient silencing of genes encoding these proteins, although depletion of IKKA led to upregulated mRNA levels of VPS28 (Fig. S7A) and lack of ESCRT-I caused increased IKKA mRNA abundance (Fig. S7B). Cells lacking ESCRT-I at 4 dpt exhibited a very potently induced expression of a gene encoding the proinflammatory cytokine IL-8 (Fig. 5B), a transcriptional target of NFκB [58]. Its mRNA levels rose in cells lacking TSG101 by around 500-fold and in cells lacking VPS28 by roughly 1000-fold with respect to control cells (Fig. 5B). Removal of IKKA prevented this strong increase (Fig. 5B), although the elevated levels of IL-8 mRNA were still observed in cells lacking IKKA and TSG101 (by 25-fold) or VPS28 (by 120-fold with respect to control cells). Importantly, although the lack of IKKA had no effect on the expression of amino acid or fatty acid oxidation genes in control cells, it partially prevented their reduced expression due to lack of ESCRT-I (Fig. 5C, top panel).

To verify the involvement of only the canonical NFκB pathway in the regulation of oxidative gene expression upon ESCRT-I depletion, we inactivated this pathway in control or ESCRT-I-deficient cells by simultaneous co-depletion of RELA. As it was the case for IKKA, removal of RELA upregulated mRNA levels of VPS28 (Fig. S7C) and lack of ESCRT-I caused increased RELA mRNA abundance (Fig. S7D). Moreover, similarly to IKKA depletion, removal of RELA strongly prevented the elevated levels of IL-8 mRNA (Fig. 5D) and partially prevented the reduced mRNA levels of most oxidative enzymes observed upon TSG101 or VPS28 depletion (Fig. 5E, top panel).

In contrast to what we observed for amino acid or fatty acid oxidation genes, depletion of IKKA or RELA did not have a clear impact on the expression of genes encoding glycolytic enzymes upon ESCRT-I deficiency (Fig. 5C and E, bottom panels). Only the elevated expression of PFKFB3 due to TSG101 or VPS28 depletion was modestly mitigated by simultaneous depletion of IKKA (Fig. 5C, bottom panel) or RELA (Fig. 5E, bottom panel).

Cumulatively, these results showed that the activation of the canonical NFκB pathway contributes to the reduced expression of amino acid or fatty acid oxidation genes but is not responsible for the induced expression of the glycolytic metabolism genes upon ESCRT-I deficiency.

### Activation of JNK signaling contributes to induced expression of glycolytic genes in cells lacking ESCRT-I

**Fig. 6:**
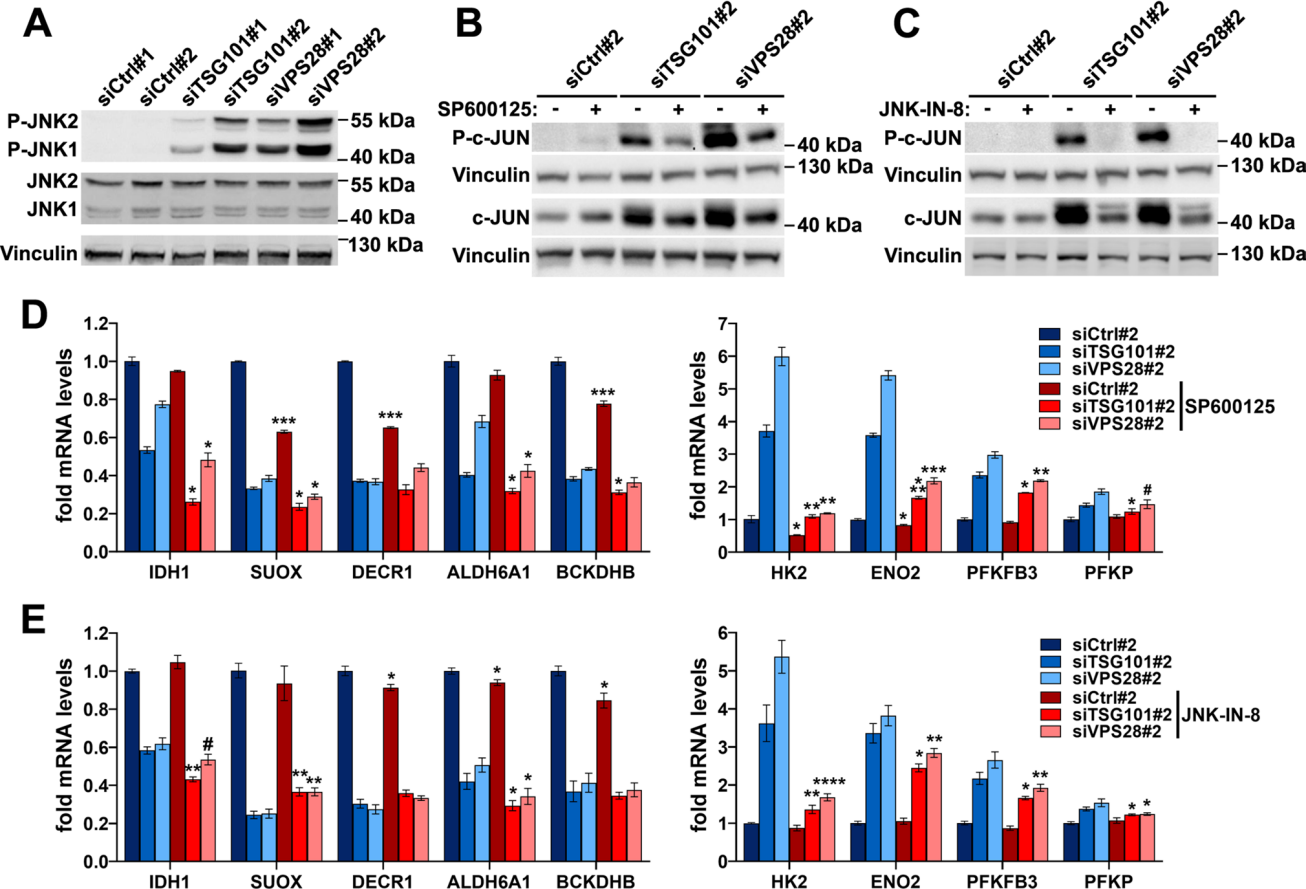
The induced expression of glycolytic metabolism genes in cells lacking ESCRT-I occurs in part due to activation of JNK signaling. **A** Western blots, performed on the same samples as blots presented in Fig. 1A, showing the levels of phosphorylated and total JNK1 and JNK2 proteins in cells depleted of ESCRT-I using two single siRNAs for each component, as compared to control cells. The analysis was performed at 3 dpt with vinculin used as a gel loading control. **B**–**C** Western blots showing the effects of ESCRT-I deficiency (siTSG101#2 or siVPS28#2) and/or inhibiting JNK kinases using SP600125 (in B) or JNK-IN-8 (in C) compounds on phosphorylation and total levels of c-JUN transcription factor**.** The analysis was performed at 3 dpt with vinculin used as a gel loading control. **D**–**E** qPCR results showing the expression of genes encoding the indicated oxidative (left graphs) or glycolytic (right graphs) enzymes at 4 dpt in control cells (siCtrl#2) or cells lacking ESCRT-I, upon 72 h treatment with DMSO or 50 μM SP600125 compound (in D) or 2 μM JNK-IN-8 compound (in E). Mean values (*n* = 3 ± SEM) are presented. Statistical significance tested by comparing the results for inhibitor-treated siCtrl#2, siTSG101#2 or siVPS28#2 with results for respective DMSO-treated cells. ^#^*P* < 0.1, *P < 0.05, **P < 0.01, ***P < 0.001

We further reasoned that the induced expression of glycolytic genes in cells lacking ESCRT-I could occur due to activation of stress response pathways other than NFκB. Glycolysis can be stimulated by the JNK signaling [36, 59], that was found to be induced upon ESCRT inactivation [32, 33]. To verify whether depletion of TSG101 or VPS28 induces JNK signaling, we analyzed by western blotting the effect of removing these proteins on phosphorylation of JNK1/2 kinases in HEK293 cells. Although undetectable in control cells, we observed a strong JNK1/2 phosphorylation in cells lacking TSG101 or VPS28 (Fig. 6A).

To investigate the involvement of JNK signaling in the transcriptional shift from oxidative to glycolytic metabolism upon ESCRT-I depletion, we treated control or ESCRT-I-deficient cells with two inhibitors of JNK kinases, SP600125 or JNK-IN-8, separately. These compounds did not affect the depletion efficiencies of TSG101 or VPS28 (Fig. S7E–F) but prevented the phosphorylation of JNK kinase target c-JUN (Fig. 6B–C) and the increase of this transcription factor abundance (Fig. 6B–C) that occurs upon JNK signaling activation [60]. Interestingly, at the applied dose, SP600125 compound seemed to be less effective than JNK-IN-8 in inhibiting c-JUN phosphorylation (Fig. 6B–C). However, both compounds were equally efficient in preventing the strong activation of the IL-8 gene expression upon ESCRT-I deficiency (Fig. S7G-H) that, in addition to NFκB, can be also induced by JNK signaling [61, 62]. Overall, the two inhibitors did not prevent the reduced expression of the analyzed amino acid or fatty acid oxidation genes upon TSG101 or VPS28 depletion, although SP600125 caused lower mRNA levels of SUOX, DECR1 and BCKDHB in control cells (Fig. 6D–E, left panels). However, both SP600125 and JNK-IN-8 partially prevented the induction of glycolytic gene expression in ESCRT-I-deficient cells (Fig. 6D–E, right panels).

The above results showed that in cells lacking ESCRT-I, the activation of JNK signaling contributes to the observed metabolic reprogramming by activating the expression of glycolytic genes.

### Impaired lysosomal degradation partially explains the metabolic reprogramming upon ESCRT-I deficiency

**Fig. 7:**
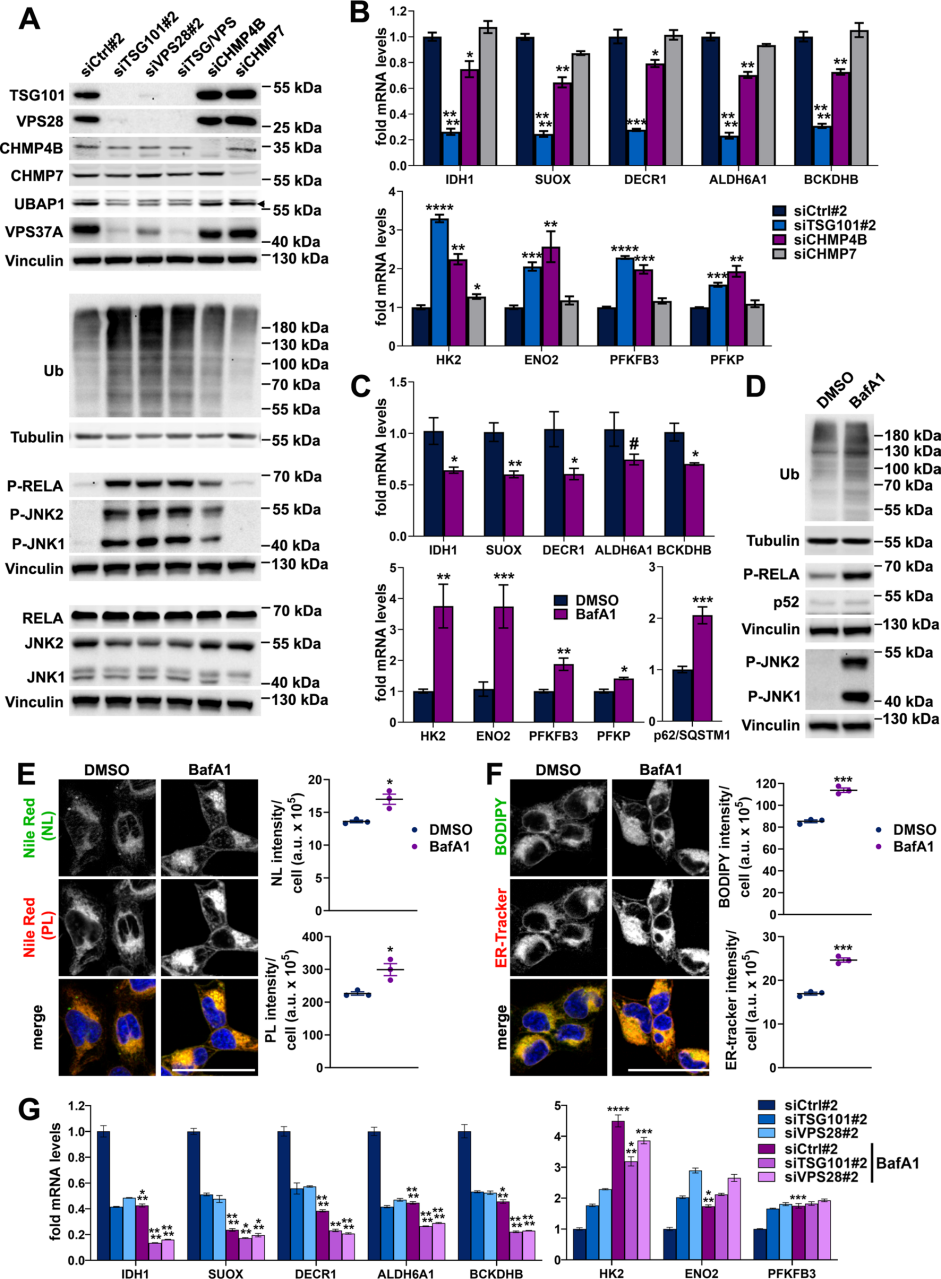
The impaired lysosomal degradation may partially explain metabolic changes in cells lacking ESCRT-I. **A** Western blots showing the levels of indicated ESCRT components, mono- and polyubiquitinated proteins (Ub) and phosphorylated as well as total RELA and JNK proteins in HEK293 cells after siRNA-mediated removal of ESCRT-I, by single depletion (siTSG101#2, siVPS28#2) or co-depletion (siTSG/VPS) of TSG101 and VPS28, or removal of ESCRT-III components (siCHMP4B or siCHMP7), as compared to control cells (siCtrl#2). The analysis was performed at 3 dpt with vinculin or tubulin used as gel loading controls. **B**–**C** qPCR results showing the effects of depleting various ESCRT components (siTSG101#2, siVPS28#2 or siCHMP4B) at 4 dpt (in B) or of 24 h treatment with 20 nM Bafilomycin A1 (BafA1; in C) on the expression of genes encoding the indicated oxidative (top) or glycolytic (bottom) enzymes and p62/SQSTM1 autophagic regulator (only in C) as compared to control cells, treated with siCtrl#2 (in B) or DMSO (in C). Mean values (*n* = 3, in B or *n* = 4, in C ± SEM) are presented. Statistical significance tested by comparison to control cells. **D** Western blots showing the levels of phosphorylated RELA, JNK1 and JNK2 proteins and levels of total p52 protein as well as mono- and polyubiquitinated proteins (Ub) in cells treated for 24 h with DMSO or 20 nM BafA1. Vinculin or tubulin used as gel loading controls. **E**–**F** Maximum intensity projection confocal images, showing the intracellular distribution of neutral lipids—NL (green), or phospholipids—PL (red) stained with Nile Red dye (shown in E) as well as the intracellular distribution of NLs stained with BODIPY 493/503 and the ER stained with ER-tracker Red (green or red, respectively in F) in live cells treated for 24 h with DMSO or 20 nM BafA1. Cell nuclei marked with Hoechst 33342 stain (blue). Scale bar, 50 μm. The dot plots on the right show total fluorescence intensities per cell (expressed in arbitrary units, a.u.). Values derived from independent experiments (dots) and their means (*n* = 3 ± SEM) are presented. Average number of cells analyzed per condition was 766 for DMSO and 561 for BafA1. **G** qPCR results showing the expression of genes encoding the indicated oxidative (left) or glycolytic (right) enzymes in cells after siRNA-mediated removal of TSG101 or VPS28 (at 4 dpt), treated for 24 h with DMSO or 20 nM BafA1, as compared to control cells (treated with non-targeting siRNA, Ctrl#2 and DMSO). Mean values (*n* = 3 ± SEM) are presented. Statistical significance tested by comparing the results for BafA1-treated siCtrl#2, siTSG101#2 or siVPS28#2 with results for respective DMSO-treated cells. ^#^*P* < 0.1, *P < 0.05, **P < 0.01, ***P < 0.001, ****P < 0.0001

Given that upon ESCRT inactivation many signaling pathways are induced due to impaired lysosomal degradation [24], we hypothesized that inhibited delivery of cargo to lysosomes could underlie the transcriptional reprogramming of cell metabolism upon ESCRT-I depletion. In accordance with this reasoning, we found that siTSG101#1, that failed to strongly induce stress response pathways (shown in Figs. 5A and 6A) and transcriptional reprogramming of cell metabolism (in Fig. 1D–F and S4B), did not cause intracellular accumulation of ubiquitinated proteins at 3 dpt, which we clearly observed by western blotting for siTSG101#2 and upon VPS28 depletion (Fig. S8A). On a side note, as compared to single depletions with siTSG101#2 or VPS28#2, simultaneous depletion using these two siRNAs did not augment ESCRT-I destabilization (judged by similarly reduced levels of UBAP1 and VPS37A ESCRT-I components) and did not aggravate the ubiquitin accumulation and JNK/RELA phosphorylation at 3 dpt (Fig. 7A and S8B, top panels) or the changes in transcription of metabolic genes at 4 dpt (Fig. S8C).

Next, we analyzed whether the transcriptional reprogramming occurs in cells lacking components of ESCRT-III, CHMP4B, that is involved in ESCRT-I-dependent delivery of cargo for lysosomal degradation [30], or CHMP7, that is specifically involved in the repair of the nuclear envelope [63, 64]. By western blotting analysis at 3 dpt, we found that depletion of CHMP4B or CHMP7 in HEK293 cells did not affect the levels of ESCRT-I components (Fig. 7A and S8B, top panels). However, lack of CHMP4B led to intracellular accumulation of ubiquitin and activation of JNK/NF-κB signaling pathways (Fig. 7A and S8B, bottom panels), although these effects were not as strong as upon depletion of TSG101 or VPS28. Conversely, depletion of CHMP7 did not cause accumulation of ubiquitin or activation of the stress-response pathways (Fig. 7A and S8B, bottom panels). By qRT-PCR analysis at 4 dpt, we observed that lack of CHMP4B but not of CHMP7 caused a reduced expression of oxidative metabolism genes (Fig. 7B, top panel) and upregulated expression of genes encoding glycolytic enzymes (Fig. 7B, bottom panel). Interestingly, whereas the expression of glycolytic genes was induced upon CHMP4B depletion to the similar extent as in cells lacking TSG101 (Fig. 7B, bottom panel), the reduction of oxidative metabolism gene expression was not as strong (Fig. 7B, top panel).

To address further whether the transcriptional reprogramming towards glycolytic metabolism in cells lacking ESCRT-I could be caused by impaired lysosomal degradation, we tested the effects of inhibiting this process using bafilomycin A1 (BafA1). We confirmed the efficiency of BafA1 treatment by qRT-PCR analysis of the expression of gene encoding p62/SQSTM1 protein (Fig. 7C, bottom panel), that is activated upon lysosomal dysfunction [65]. Importantly, BafA1 led to reduced expression of all tested amino acid or fatty acid oxidation genes (Fig. 7C, top panel) and increased the expression of all tested glycolytic metabolism genes (Fig. 7C, bottom panel). These changes, as in the case of ESCRT-I deficiency, were associated with the accumulation of ubiquitinated proteins and the increased phosphorylation of RELA and JNK1/2 (Fig. 7D). Interestingly, BafA1 had no effect on the levels of p52 transcription factor (Fig. 7D), indicating the activation of canonical but not non-canonical NF-κB signaling. Moreover, by confocal microscopy, we observed that, likewise ESCRT-I deficiency (shown in Fig. 2C–D), BafA1 led to intracellular accumulation of NLs and PLs (Fig. 7E–F) and to the elevated ER content (Fig. 7F).

Finally, we investigated the effects of BafA1 treatment on metabolic gene expression upon TSG101 or VPS28 depletion at 4 dpt. We found that the reduction of the oxidative gene expression was stronger in BafA1-treated ESCRT-I-deficient cells than in DMSO-treated ESCRT-I-deficient cells or BafA1-treated control cells (Fig. 7G, left panel). This additive effect of BafA1 and ESCRT-I deficiency on the expression of the oxidative metabolism genes, indicated that these treatments may regulate their expression by distinct mechanisms. However, we did not observe such additive effects on the expression of genes encoding glycolytic enzymes (Fig. 7G, right panel). The mRNA levels of ENO2 and PFKFB3 upon ESCRT-I deficiency were not further increased by BafA1 treatment and the induction of *HK2* gene expression upon BafA1 treatment was not intensified by ESCRT-I deficiency (Fig. 7G, right panel). Hence, we concluded that ESCRT-I deficiency and BafA1 may affect the expression of genes encoding glycolytic enzymes by similar mechanisms.

**Fig. 8:**
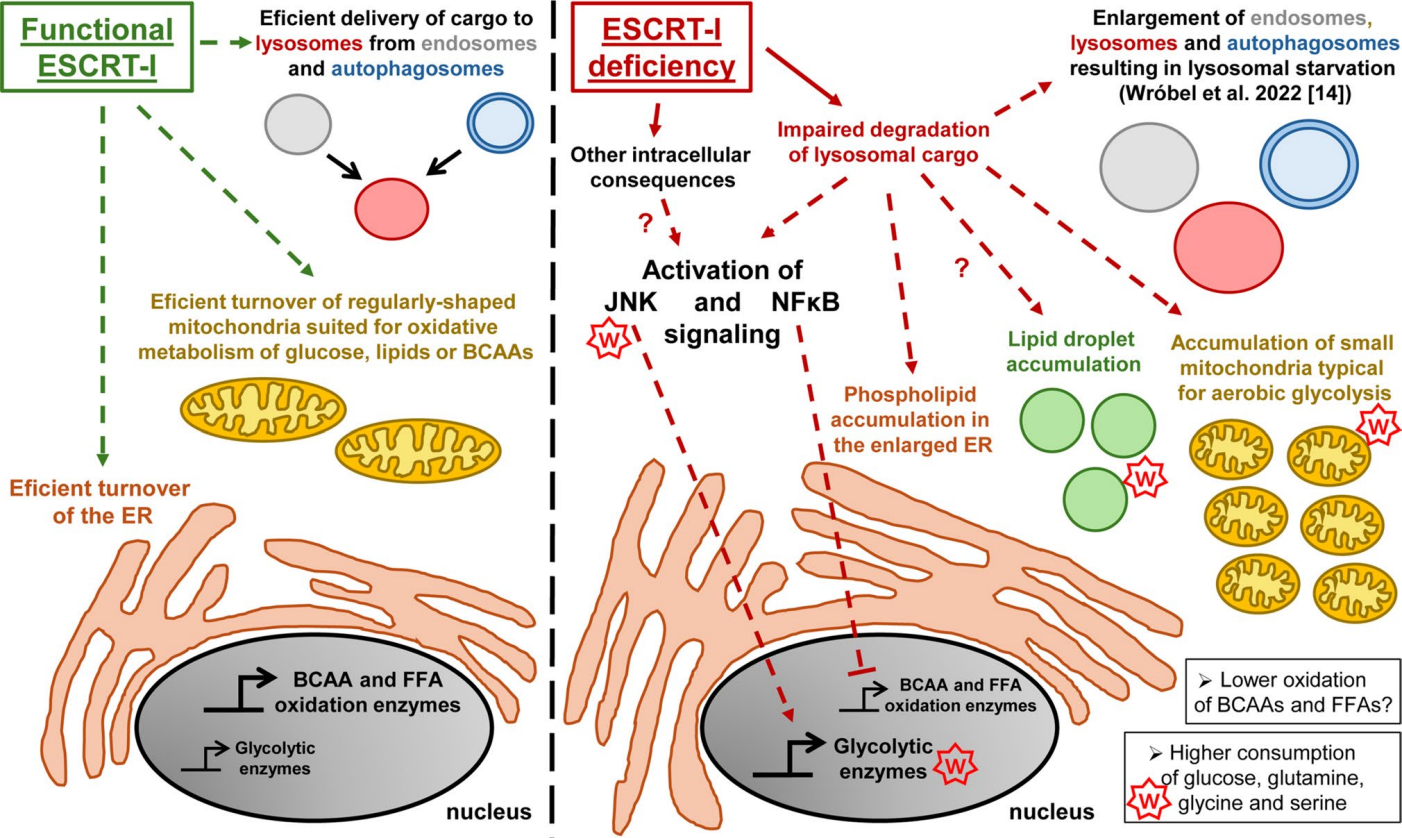
Graphical summary depicting the role of functional ESCRT-I and consequences of its deficiency on organelle homeostasis and cell metabolism. Dashed arrows indicate processes regulated by ESCRT-I (in green) or consequences of ESCRT-I deficiency (in red), including the involvement of JNK and NFκB signaling pathways in regulating the expression of genes encoding enzymes involved in oxidation of free fatty acids (FFA) or branched-chain amino acids (BCAA) as well as glycolysis. The “W” signs underscore the consequences of ESCRT-I deficiency that are established hallmarks of the Warburg effect. Question marks indicate the relationships that remain to be clarified

Collectively, by analyzing cells with different ways of ESCRT inactivation and cells treated with BafA1, we found that upon ESCRT-I dysfunction, transcriptional regulation of oxidative or glycolytic gene expression is associated with intracellular ubiquitin accumulation and at least in part it occurs due to inhibition of lysosomal degradation (Fig. 8).

## Discussion

Collectively, our results show that functional ESCRT-I promotes oxidative metabolism of nutrients, such as branched-chain amino acids or fatty acids, over aerobic glycolysis, by restricting stress and inflammatory pathways, potentially due to the involvement of this complex in delivering cargo for lysosomal degradation (Fig. 8). A metabolic switch towards aerobic glycolysis occurs during many important cellular processes, such as somatic cell reprogramming, macrophage polarization, tissue remodeling during inflammation and carcinogenesis [8, 66–68] but still many of its underlying aspects are not well understood. A growing number of studies has shown that endocytic trafficking affects cell metabolism [69, 70] and that particular endocytic machineries may regulate oxidative or glycolytic metabolism [71–73]. Hence, it is likely that endocytic proteins are involved in metabolic reprogramming events. Here we provide multiple pieces of evidence that inactivation of ESCRT-I leads to metabolic reprogramming closely resembling the Warburg effect (Fig. 8).

By transcriptomic analysis and its validation, we discovered that removal of ESCRT-I in HEK293 or HepG2 cells leads to opposite regulation of the expression of two particular groups of metabolism-related genes: those involved in oxidative metabolism of carboxylic acid-containing nutrients (with downregulated expression) and those related to glycolysis (with upregulated expression). The group of downregulated genes includes those encoding enzymes that mediate initial reactions of oxidative breakdown of FFAs and BCAAs. These reactions provide esters of CoA and electron carriers, such as NADH, thus molecules that are incorporated into further oxidation in mitochondria within the citric acid cycle and oxidative phosphorylation [2, 74, 75]. Hence, although the metabolism-related genes, whose expression is reduced upon ESCRT-I depletion, do not encode key mitochondrial components, they function in obtaining energy from oxidation of specific nutrients, such as FFAs and BCAAs.

The group of upregulated genes implicated in glycolysis, although small, encompasses those encoding rate-limiting glycolytic enzymes, HK2, ENO2 and PFKP, as well as an enzyme that potently stimulates glycolysis, PFKFB3 [76, 77]. Consistently with upregulated expression of these genes, we discovered that cells lacking ESCRT-I have elevated lactate production and increased glycolysis-related ECAR, hence they perform aerobic glycolysis [1]. Moreover, we discovered that changes in metabolic gene expression observed upon ESCRT-I deficiency are associated with elevated consumption of glucose and of amino acids, known to be important for cells with the Warburg effect [9–11]. Among amino acids particularly used by cells lacking ESCRT-I is glutamine, whose elevated oxidation in mitochondria could explain high ATP-linked respiration of cells lacking ESCRT-I. Hence, we propose that ESCRT-I deficiency leads to preferential usage of glucose and glutamine over FFAs and BCAAs for energy production (Fig. 8).

The here reported reduced expression of amino acid and fatty acid oxidation genes upon ESCRT-I dysfunction coincides with a broad accumulation of intracellular membranes. As we described previously, cells lacking ESCRT-I have enlarged endosomes and lysosomes, that are hallmarks of inhibited endolysosomal degradation [14, 30]. In the current study we also show that these cells accumulate mitochondria and lipid droplets, and their ER is expanded (Fig. 8). The increased abundance of mitochondria and the enlarged ER in cells lacking TSG101 or VPS28 are likely due to impaired autophagic degradation of these organelles [26, 78]. Whether the accumulation of mitochondria occurs due to the impaired removal of damaged ones, requires further investigation. The ER expansion could also be a consequence of its increased biogenesis, for instance to produce a branched network of peripheral tubular structures [79], that could be addressed in detail by electron microscopy. It could also be caused by reduced delivery of the ER membrane parts to other organelles, including the Golgi, lysosomes or autophagosomes, although it is less likely, given the overall enlargement of these organelles upon ESCRT-I deficiency [14]. However, as ESCRT machinery has recently been implicated in the ER-to-Golgi transport [80], potential inhibition of this process upon ESCRT-I deficiency may also contribute to the ER enlargement. Accumulation of lipid droplets could be a result of the observed here changes in metabolic gene expression but also of diminished lipid transport from the droplets into mitochondria proposed to be mediated by ESCRT-I proteins [81]**.**

The profoundly impaired turnover of organelles and intracellular membranes in the absence of ESCRT-I likely results in a reduced supply of molecules that are recycled from lysosomal degradation, including FFAs. Our finding that cells lacking ESCRT-I exhibit not reduced but even slightly elevated FFA levels allows hypothesizing that transcriptional regulation of genes involved in FFA metabolism and synthesis in these cells compensates for reduced fatty acid delivery from lysosomes. Similarly, although ESCRT-I-deficient cells have impaired degradation of proteins (likely membrane proteins and components of non-degraded organelles), our ^1^H-NMR analysis showed that these cells do not suffer from a general shortage of intracellular free amino acids. This could be in part due to observed by us elevated consumption of extracellular amino acids, such as glutamine, glycine or serine, and in part due to potentially reduced mitochondrial oxidation of other amino acids, particularly BCAAs. Further experiments should address whether oxidation of fatty acids or BCAAs is indeed reduced upon ESCRT-I deficiency.

In this study, we corroborated our previous observation that lack of ESCRT-I does not affect the general mTORC1 signaling [14]. Therefore, we reasoned that the observed changes in the expression of metabolic genes are not due to general regulation of mTOR kinase activity. However, we were intrigued by the finding that the reduced expression of genes related to oxidative metabolism occurs in cells in which we previously observed a prominent activation of TFEB/TFE3 transcription factors [14] that may promote expression of genes related to fatty acid oxidation [38]. By addressing this subject, we clarified that the activation of TFEB/TFE3 transcription factors partially prevents the reduced expression of genes related to fatty acid and amino acid oxidation. The activation of TFEB/TFE3 factors serves as a homeostatic response in an attempt to rescue the impaired lysosomal degradation [65]. Hence, a stronger inhibition of expression of amino acid and fatty acid oxidation genes upon ESCRT deficiency in cells lacking TFEB/TFE3 is consistent with our interpretation that the changes in metabolic gene expression in cells lacking ESCRT-I may be due to inhibited lysosomal degradation.

Importantly, we demonstrate that activation of the NFκB pathway in mammalian cells lacking ESCRT-I, in addition to promoting inflammatory signaling, inhibits the expression of genes encoding enzymes of amino acid or fatty acid oxidation. This effect may be indirect, e.g. due to NFκB-dependent regulation of other transcription factors as NFκB signaling may affect transcriptional regulators of metabolism such as PPAR nuclear receptors or SIRT1 deacetylase [34, 57]. The role of the NFκB signaling in regulation of glycolytic metabolism is not well established. Some reports suggest that it promotes [35, 82] and some others that it inhibits glycolysis [83, 84]. We observe that preventing the activation of NFκB signaling does not interfere with increased expression of glycolytic genes upon ESCRT-I deficiency.

Our findings that ESCRT-I deficiency in mammalian cells leads to activation of the JNK pathway extend similar observations made previously for mutants of genes encoding ESCRT-0 or ESCRT-II components [32, 33]. Whereas those studies showed that the activation of JNK signaling may affect cell proliferation or survival upon ESCRT dysfunction [32, 33], we demonstrate that activation of this signaling participates in inducing the expression of glycolytic genes in cells lacking TSG101 or VPS28. Intriguingly, the exact three glycolytic genes with the highest upregulation upon ESCRT-I deficiency (encoding HK2, ENO2 and PFKFB3) were reported to have increased expression in cancer-associated fibroblasts due to activation of JNK signaling upon compression-induced stress [59]. Hence, these genes appear to be a part of a particular JNK-dependent transcriptional response commonly activated upon various stress conditions. Further investigation is required to unravel which transcription factors mediate this response downstream of JNK kinases in cells lacking ESCRT-I.

As we observe that NFκB and JNK signaling pathways only partially contribute to the transcriptional reprogramming of cell metabolism due to ESCRT-I deficiency, it is possible that some other pathways could be also involved. ESCRTs have been shown to regulate ERK1/2 and WNT/β-catenin signaling [24] that are known to mediate metabolic reprograming [36, 85]. Moreover, the reprogramming could also be a consequence of NFκB/JNK-independent activation or inhibition of transcription factors such as KLF15 or PPARβ/δ that control oxidative and/or aerobic metabolism [86, 87]. However, most likely, lack of ESCRT-I affects multiple pathways with redundant roles in regulation of cell metabolism, thus deciphering precise mechanisms may be very challenging.

Our hypothesis that the metabolic shift in cells lacking ESCRT-I may occur due to impaired lysosomal degradation is supported by the results showing association between intracellular ubiquitin accumulation, the activation of stress response pathways and transcriptional reprogramming towards glycolytic metabolism. Although it has been previously proposed that pharmacological inhibition of lysosomal degradation may promote glycolytic metabolism [88], to our knowledge the effects of such treatment on the expression of genes related to glycolytic or oxidative metabolism have not been demonstrated. Here we show that BafA1 causes a transcriptional reprogramming from amino acid and fatty acid oxidation to glycolytic metabolism, similarly to what we observe in cells lacking ESCRT-I. Most importantly, no additive effect of BafA1 treatment and TSG101 or VPS28 depletion on activation of glycolytic gene expression confirms that at least in part the metabolic reprogramming upon ESCRT-I deficiency is a consequence of impaired lysosomal degradation. However, we observed a clear additive effect on the reduction of oxidative gene expression between BafA1 treatment and ESCRT-I removal. Hence, although we provide new insights into the relation between ESCRT-I-dependent lysosomal degradation and regulation of cell metabolism, still some important aspects of this relation remain elusive.

Overall, the results of our study suggest that ESCRT-I activity could potentially promote oxidative metabolism, while its inactivation could favor glycolysis. Further studies should address whether the abundance and/or activity of ESCRT-I is regulated upon stem cell differentiation that involves reprogramming from glycolysis to oxidative metabolism [89–91], or upon inducing cell pluripotency, inflammation or oncogenesis that involve a reprogramming from oxidative to glycolytic metabolism [8, 66–68].

## Materials and methods

### Antibodies

The following antibodies, with indicated catalogue numbers (Cat#), and working dilutions for western blotting (WB) or immunofluorescence (IF) were used: anti-TSG101 (Cat# ab83, 1:500 in WB), anti-VPS28 (Cat# ab167172, 1:500 in WB), anti-TOM20 (Cat# ab186734, 1:200 in IF) and anti-SUOX (Cat# ab129094, 1:1000 in WB) from Abcam; anti-RELA (Cat# 6956, 1:1000 in WB), anti-P-RELA (Cat# 3033, 1:500 in WB), anti-p52 (Cat# 4882, 1:2000 in WB), anti-P-S6 (Cat# 2211, 1:2000 in WB), anti-JNK1/2 (Cat# 9252, 1:1000 in WB) and anti-P-JNK1/2 (Cat# 9255, 1:1000 in WB) from Cell Signaling Technologies; anit-IDH1 (Cat# GT1521, 1:1000 in WB) from GeneTex; anti-ALDH6A1 (Cat# sc-271582, 1:500 in WB), anti-HK2 (Cat# sc-374091, 1:1000 in WB), anti-P–c-JUN (Cat# sc-822, 1:1000 in WB) and anti-c-JUN (Cat# sc-44, 1:1000 in WB) from Santa Cruz; anti-PFKFB3 (Cat# 13,763-1-AP, 1:1000 in WB), anti-VPS37A (Cat# 11,870-1-AP, 1:1000 in WB) and anti-CHMP4B (Cat# 13,683-1AP, 1:1000 in WB) from Proteintech; anti-CHMP7 (Cat# HPA036119, 1:500 in WB) from Atlas Antibodies; anti-UBAP1 (Cat# NBP2-58,969, 1:1000 in WB) from Novus Biologicals, anti-mono- and -polyubiquitinylated conjugates (Cat# BML-PW8810, 1:1000 in WB, 1:100 in IF) from Enzo Life Sciences; anti-CLNX (Catt# C4731, 1:100 in IF), anti-tubulin (Cat# T5168, 1:4000 in WB) and anti-vinculin (Cat# V9131, 1:4000 in WB) from Sigma-Aldrich; secondary horseradish peroxidase (HRP)-conjugated goat anti-mouse and goat anti-rabbit from Thermo Fisher Scientific.

### Plasmids

pLenti-CMV-MCS-GFP-SV-puro (Addgene plasmid #73,582) was a gift from Katarzyna Mleczko-Sanecka. psPAX2 (Addgene plasmid #12,260) and pMD2.G (Addgene plasmid #12,259) lentiviral packaging plasmids were a gift from Didier Trono.

### Cell culture and treatment

HEK293 and HEK293T embryonic kidney cells were maintained in Dulbecco’s modified Eagle’s medium (DMEM, Sigma-Aldrich, M2279). HepG2 hepatoblastoma cells were cultured in Eagle’s minimum essential medium (EMEM, ATCC, 30–2003). Both medium types were supplemented with 10% (v/v) fetal bovine serum (FBS, Sigma-Aldrich, F7524) and 2 mM L-Glutamine (Sigma-Aldrich, G7513). All cell lines were regularly tested as mycoplasma-negative and their identities were confirmed by short tandem repeat (STR) profiling performed by the ATCC Cell Authentication Service.

Bafilomycin A1 (Sigma-Aldrich, B1793) at 20 nM concentration was applied for 24 h to inhibit lysosomal degradation. To inhibit JNK activity, SP600125 (MedChemExpress, HY-12041) was used at 50 μM concentration and JNK-IN-8 (MedChemExpress, HY-13319) was used at 2 μM concentration, both for 72 h. To inhibit mTOR kinase activity INK128 (Selleckchem, S2811) was used at 100 nM concentration for 48 h.

### Cell transfection and lentiviral transduction

HEK293 cells were seeded on 6-well (1 × 10^5^ cells/well) or 12-well (1 × 10^5^ cells/well) plates for western blotting and quantitative real-time PCR (qRT-PCR) experiments or on 0.2% gelatin (Sigma Aldrich, G1890)-covered 96-well plate (Grainer Bio-One, 655–090) (2.5 × 10^3^ cells/well) for confocal microscopy. HepG2 cells were seeded on 6-well plates (2 × 10^5^ cells/well) for western blotting or on gelatin-covered 96-well plates (4 × 10^3^ cells/well) for microscopy. 24 h after seeding, cells were transfected with 20 nM siRNAs using Lipofectamine™ RNAiMAX Transfection Reagent (Thermo Fisher Scientific, 13,778,150) according the manufacturer’s instructions and imaged or harvested after 3 or 4 days post transfection (dpt). The following Ambion Silencer Select siRNAs (Thermo Fisher Scientific) were used: Negative Control No. 1 (siCtrl#1, 4,390,843), Negative Control No. 2 (siCtrl#2, 4,390,846) and Negative Control No. 3 (siCtrl#3, custom-made, UACGACCGGUCUAUCGUAG); siTsg101#1 (s14439), siTsg101#2 (s14440), siVps28#1 (s27577), siVps28#2 (s27579), siTFEB (s15496), siTFE3 (s14031), siIKKA (s3076), siRELA (s11916), siDRP1 (s19560), siOPA1 (s9852), siCHMP4B (s43363), siCHMP7 (s40781). In experiments with simultaneous knockdown of two genes, the total concentration of siRNA was adjusted to 40 nM using siCtrl#2.

For CRISPR/Cas9-mediated knock-out of the gene encoding TSG101, 2 control non-targeting gRNA sequences and 1 targeting gRNA sequence [92] were cloned into the lentiCRISPRv2 vector. Lentiviral particles were produced in HEK293T cells using packaging plasmids: psPAX2 and pMD2.G, as described elsewhere [93]. Subsequently, 1 × 10^6^ HepG2 cells were grown in 5 ml of virus-containing EMEM medium on P60 dish for 1 h. After the infection, the cells were kept in fresh EMEM for 24 h. Then, cells were split and grown in selection EMEM medium containing 1 µg/ml puromycin for 7 days. The sequences of gRNAs used in this study are: gCtrl#1- CGCTTCCGCGGCCCGTTCAA, gCtrl#2- CTGAAAAAGGAAGGAGTTGA, gTsg101#1- AGGGAACTAATGAACCTCAC.

### Microarray analysis

HEK293 cells were transfected with control (siCtrl#1 or siCtrl#2) or ESCRT-I-targeting (siTSG101#1, siTSG101#2, siVPS28#1 or siVps28#2) siRNAs in three biological repetitions. Three days post transfection (3 dpt) cells were collected and cRNA was prepared as described elsewhere [94]. cRNA samples hybridized to the Human HT-12 BeadChip array (Illumina) were scanned on the BeadArray Reader (Illumina) and analyzed using BeadStudio v3.0 (Illumina) and BeadArray R package v1.10.0 software according to a procedure described elsewhere [29]. Upon quantile normalization, data were log2 transformed and statistical analysis of the results was performed using a t-test followed by false discovery rate (FDR) correction for multiple testing according to the Benjamini and Hochberg method.

Ingenuity Pathway Analysis software was used to identify canonical pathways potentially activated or inhibited based on the common list of genes whose expression was significantly increased or decreased (FDR < 0.05) in cells lacking TSG101 or VPS28. GO analysis of biological processes or cellular components was performed using enrichGO function from clusterProfiler R‐package (version 4.10.0; [95]), on the list of the genes with commonly downregulated expression (FDR < 0.05, log2 fold change to controls ≤ − 0.6). Heatmaps were plotted using ComplexHeatmap (version 2.16.0, [96]). The visualizations were performed in R version 4.3.1 (https://www.R-project.org). Heatmaps for genes encoding enzymes of glucose catabolism and proteins involved in biosynthesis of FFAs, TGs or PLs visualize the expression of genes from Gene Ontology lists (glycolytic process_GO:0006096, acetyl − CoA biosynthetic process from pyruvate (GO:0006086), fatty acid biosynthetic process_GO:0006633, triglyceride biosynthetic process_GO:0019432, glycerophospholipid biosynthetic process_GO:0046474) that were manually curated to remove unrelated genes.

### Quantitative real-time PCR (qRT-PCR)

Total RNA was isolated from cells using High Pure Isolation Kit (Roche, 11,828,665,001) according to the manufacturer’s instruction. For cDNA preparation, 1000 ng of total RNA, random nonamers (Sigma-Aldrich, R7647), oligo(dT)23 (Sigma-Aldrich, O4387) and M-MLV reverse transcriptase (Sigma-Aldrich, M1302) were used. In most qRT-PCR analyzes, the NCBI Primer designing tool was used to design primers that were custom-synthesized by Sigma-Aldrich (primer sequences listed in Table 1) and cDNA sample amplification was performed with the KAPA SYBR FAST qPCR Kit (KapaBiosystems, KK4618). Only to analyze the expression of amino acid or fatty acid oxidation genes at 3 days post transfection (shown in Fig. 1E), TaqMan™ assays (ThermoFisher Scientific, 4,331,182) with TaqMan™ Gene Expression Master Mix (ThermoFisher Scientific, 4,369,016) were used (assays listed in Table 2). In both, using custom-synthesized primers and TaqMan™ assays, the 7900HT Fast Real-Time PCR thermocycler (Applied Biosystems) was used for DNA amplification. Data were normalized according to the expression level of the housekeeping genes encoding ACTB (β-actin) or HPRT1 (Hypoxanthine Phosphoribosyltransferase 1) proteins. Results are presented as fold changes compared to the average values obtained for control cells or to values obtained using siCtrl#2 (in co-depletion experiments).

**Table 1:** Sequences of primers used for qRT-PCR

Transcript name	Forward primer	Reverse primer
ACTB	CAGGTCATCACCATTGGCAAT	TCTTTGCGGATGTCCACGT
HPRT1	AGTCCTATTGACATCGCCAG	TAGGAATGCAGCAACTGACA
ALDH6A1	GACCAACCATCATCTCGAA	TGGCTTCATCCAATGTTTCT
BCKDHB	CATTTTACTTTCCAGCCAGATCC	AGGCAACATCTTCACCAAAT
DECR1	GCGATGCTACCACCTAATAGT	TATCACGCACTGAGCACCTA
IDH1	TGACACGAATCATTTGGGAA	TGGCATCACGATTCTCTATG
SUOX	GACCCTATTAGGTCTCGGT	GGAACTCACTTCCTCCTTAG
HK2	CCTCGGTTTCCCAACTCT	GAGATACTGGTCAACCTTCTGC
ENO2	TCTACCAGGACTTTGTCAGG	AATCTGGATCCCTACATTGG
PFKFB3	TGACCTACGAGGAGATCAG	CATGATCACTGGCTCCAAG
PFKP	CTATGACGTGTCGGACTCAG	AAACAGACACAGTCCACGG
IL-8	ACTCCAAACCTTTCCACCCC	TCTCAGCCCTCTTCAAAAACTTC
TSG101	CCTCCCAATCCCAGTGGTTACCCA	GGTGTCCTCGCTGATTGTGCCA
VPS28	AGCCGTCCAGGTCTCAGTGC	ATTGTCTTCACCACCGCAAAC
TFEB	GCAACAGTGCTCCCAATAG	TCAGGATTGATGTAGCCAAG
TFE3	TCCGGGATTGTTGCTGACA	GCAGTGATATTGGGAGGCTTG
IKKA	TACTTTGAAGCAGCCAAGAT	TGAAGACTTTCATCAGGTGG
RELA	AGCTTGTAGGAAAGGACTGCC	ATAGGAACTTGGAAGGGGTTGTTGT
p62/SQSTM1	GAATCAGCTTCTGGTCCATCGG	GCTTCTTTTCCCTCCGTGCT

**Table 2:** TaqMan assays used for qRT-PCR

Transcript name	Catalogue number
ACTB	Hs99999903
ALDH6A1	Hs00194421
BCKDHB	Hs00609053
DECR1	Hs00154728
IDH1	Hs01855675
SUOX	Hs04183429

### Western blotting

Cells were lysed in RIPA buffer (1% Triton X-100, 0.5% sodium deoxycholate, 0.1% SDS, 50 mM Tris pH 7.4, 150 mM NaCl, 0.5 mM EDTA) supplemented with phosphatase inhibitor cocktails 2 and 3 (Sigma-Aldrich, P5726 and P0044) and protease inhibitor cocktail (6 μg/ml chymostatin, 0.5 μg/ml leupeptin, 10 μg/ml antipain, 2 μg/ml aprotinin, 0.7 μg/ml pepstatin A and 10 μg/ml 4-amidinophenylmethanesulfonyl fluoride hydrochloride; Sigma-Aldrich). BCA Protein Assay Kit (Thermo Fisher Scientific, 23,225) was used to measure protein concentration. Subsequently, 15–25 µg of total protein per sample were resolved on 8%, 10% or 12% SDS-PAGE and transferred onto nitrocellulose membrane (Amersham Hybond, GE Healthcare Life Science, 10,600,002). Membranes were blocked in 5% milk PBS with 0.1% Tween followed by incubation with specific primary and secondary antibodies. For signal detection, Clarity Western ECL Substrate (BioRad, 170–5061) and ChemiDoc imaging system (Bio-Rad) were applied. Image Lab 6.0.1 software (Bio-Rad) was used for densitometric analysis of western blotting bands. The raw data were normalized to vinculin or tubulin band intensities and presented as fold levels to the average values obtained for control cells.

### Lipid extraction, separation and gas chromatography-mass spectrometry (GC–MS) analysis

HEK293 cells were cultured in 100 mm dishes and 3 days post siRNA transfection were harvested, washed with PBS and cell pellets were processed for lipid extraction. Lipids were extracted according to the Bligh and Dyer method [97]. Each sample was homogenized in a glass tube in chloroform:methanol (2:1) containing 0.01% (w/v) butylated hydroxytoluene (BHT). Then distilled water was added to each tube and the tubes were vortexed and centrifugated at 3000 rpm for 10 min at 4ºC. After centrifugation, the resulting two-phase system was separated and the lower phase containing lipids was collected.

Lipid extracts were separated by thin-layer chromatography on silica gel 60 plates (Merck, Darmstadt, Germany) in heptane/isopropyl ether/glacial acetic acid (60/40/4, vol/vol/vol) with authentic standards. For fatty acid analysis, bands corresponding to free fatty acids (FFAs), triglycerides (TGs), diglycerides (DAGs), and phospholipids (PLs) were visualized with 0.2% 2,3-dichlorofluorescein, scraped from the plate into screw-capped glass tubes and transmethylated in the presence of 14% boron trifluoride in methanol. The resulting FA methyl esters were extracted with hexane and subjected to Agilent 7890A-5975C GC–MS instrument with an Agilent 19091N-205 capillary column (Agilent Technologies, Santa Clara, CA, USA). Peak alignment, purity and quality analyses were performed using Agilent MSD Productivity ChemStation software. Methyl nonadecanoate was used as an internal standard for further compound quantification by selected ion monitoring. For normalization, the measured concentrations of various lipid classes (nmol/μl) were divided by protein concentration (μg/μl) that was assessed using BCA Protein Assay Kit (Thermo Fisher Scientific, 23,225).

### Confocal microscopy

For live cell imaging, cells grown on 0.2% gelatin-coated 96-well plates (Greiner Bio-One, 655–090) were incubated for 15 min with 1 μg/ml Nile Red (N1142), 0.4 μg/ml BODIPY 493/503 (D3922), 1 μM ER-tracker Red (E34250), 100 nM MitoTracker™ Deep Red FM (M22426), 100 nM TMRE (T669) and imaged immediately. For imaging of fixed cells, cells grown on gelatin-coated 96-well plates were fixed with 3.6% PFA and immunostained as described elsewhere [30]. Cell nuclei were marked with Hoechst 33,342 (Thermo Fisher Scientific, H3570) or DAPI (Sigma-Aldrich, D9542), as indicated in the figure legends. Plates were scanned using Opera Phenix high content screening microscope (PerkinElmer) with 40 × 1.1 NA water immersion objective. Harmony 4.9 software (PerkinElmer) was applied for image acquisition and their quantitative analysis. The detection of fluorescent signals with the Opera Phenix microscope was performed with settings adjusted to ensure that signals are not saturated. All the images were collected with pixel binning set to "2", with the exception of TOM20-staining that was imaged with binning set to “1”. Maximum intensity projection images were obtained from 3 z-stack planes with 1 μm interval. Pictures were assembled in ImageJ and Photoshop (Adobe) with only linear adjustments of contrast and brightness. For quantification of integral fluorescence intensities, as well as number and area of lipid droplets, more than 10 microscopic fields were analyzed per each experimental condition. The quantitative analyses using Harmony 4.9 software were performed on raw images, hence were not affected by adjustments made for visual presentation of representative images. The number of cells analyzed per condition is provided in figure legends. To analyze mitochondria morphology, the machine learning module of Harmony 4.9 software was used to find different classes of cells by analyzing texture properties of imaged TOM20 staining. The DRP1- or OPA1-depleted cells were used to train the software to recognize cells with elongated or fragmented mitochondria, respectively.

### Lactate content analysis

Colorimetric analysis of intracellular abundance of lactate was performed using Lactate Assay Kit (Sigma-Aldrich, MAK064) according the manufacturer’s instructions. For this analysis, HEK293 cells were cultured in 12-well plates and 4 days post transfection with siRNAs (4 dpt) they were harvested into assay buffer and homogenized using a tissue homogenizer. For normalization, the measured amount of lactate in the assay buffer (nmol/μl) was divided by DNA concentration (μg/μl) that was assessed using NanoDrop 1000 spectrophotometer (Thermo Fisher Scientific).

### Energy metabolism analysis

Oxygen consumption rate (OCR) as well as extracellular acidification rate (ECAR) were measured with the Seahorse XFe24 Analyzer (Agilent Technologies, Santa Clara, CA, USA). To achieve this, 4 × 10^4^ or 8 × 10^4^ cells per well were plated in Seahorse 24-well cell plates for 3 dpt (OCR) and 4 dpt (ECAR) measurements, respectively. On the next day, cells were transfected with siRNAs at 20 nM concentration. OCR and ECAR were measured according to manufacturer’s instructions by sequential injections of compounds that modulate mitochondrial function: 4 μM oligomycin (Sigma-Aldrich) that inhibits ATP-linked respiration, 2 μM carbonyl cyanide-p-trifluoromethoxyphenylhydrazone (FCCP, from Sigma-Aldrich) that allows for maximal electron flow through the electron transport chain, thus maximal oxygen consumption, and 2 μM antimycin A (Sigma-Aldrich) together with 2 μM rotenone (Sigma-Aldrich) that shut down mitochondrial respiration. To assess glycolysis-related ECAR an additional injection of 100 mM 2-deoxy-d-glucose (2-DG, from Sigma-Aldrich), an inhibitor of glycolysis, was performed. The results (pmol of consumed oxygen per minute and mpH altered per minute) were normalized to the fluorescence intensity of 10 μM Hoechst 33,342 (Thermo Fisher), added to cells during the assay, that stains DNA and reflects cell number.

### ^1^H-Nuclear magnetic resonance (^1^H-NMR) analysis of metabolites

The ^1^H-NMR analysis was used to detect polar metabolites in pellets of cells recovered from culture dishes by trypsinization at 4 dpt and in DMEM cell culture medium (fresh or after culture of cells from 3 to 4 dpt). The metabolites from pellets of 7–10 × 10^6^ cells were extracted using 500 µl of cold methanol: water (8:2, v/v) solvent. The samples were then homogenized at a frequency of 30 MHz using TissueLyser LT (QIAGEN, Germantown, USA) for 5 min, stored at − 20 °C for 20 min and centrifuged (5 min, 14,000 rpm, 4 °C). Following these steps, the calculated volume of the supernatant was transferred into a new Eppendorf tube and evaporated (1,100 rpm, 4.5 h, 40 °C). Subsequently, the dried polar extracts were dissolved in 700 μl of PBS buffer (0.1 M, 50% D_2_O, pH = 7.2, TSP = 0.03 mM) and mixed for 3 min. Finally, 600 μl of the solution was transferred into 5-mm NMR tubes for immediate measurements. The metabolites from the normalized volume of media were extracted using cold methanol:water (8:2, v/v) solvent with a ratio (4:7) sample: solvent. The samples were then processed as samples of cell pellets although with the different PBS buffer composition (0.1 M, 100% D_2_O, pH = 7.2, TSP = 0.03 mM).

The one-dimensional (1D) NMR spectra of cellular extracts were acquired with the cpmgpr1d (Bruker notation) pulse sequence by suppression of water resonance by presaturation, at 298 K using an Avance II Spectrometer (Brukrer, GmBH, Germany), operating at a proton frequency of 600.85 mHz. Acquisition parameters were as follows: spectral width, 19.82 ppm; the number of scans, 128; acquisition time, 2.72 s per scan; relaxation delay, 3.5 s; and time-domain points, 64 K. The spectra were referenced to the TSP resonance at 0.0 ppm and manually corrected for phase and baseline (MestReNova v. 14.0.2).

All obtained spectra were transferred to MATLAB (Matlab R2014a, v. 8.3.0.532, Natick, MA, USA) for preprocessing. The spectral section in the range of 4.650 to 5.159 ppm for cell pellets samples and 4.681 and 5.047 ppm for media samples, corresponding to suppression of water resonance signals, was removed from the data analyzed. The alignment procedures of resonance signals were carried out by using the correlation of optimized wrapping (COW) and interval correlation shifting icoshift algorithms. The spectra consisted of 8910 data points and were normalized using the probabilistic quotient normalization (PQN) method. The metabolites were identified using Chenomx software (v 8.4 Chenomx Inc. Edmonton, Alberta, Canada) and the online database, e.g. Human Metabolome Data Base (www.hmdb.ca) and verified with published literature (Tables S3 and S4).

Statistical analysis was performed using 22 intracellular and 30 extracellular metabolites independently. For each metabolite, a single representative signal was used, and these signals with their respective NMR assignments are detailed in the supplementary materials (Table S3 and S4). In the NMR measurements, relative integration indicates the number of protons responsible for each signal, facilitating the quantification of relative concentrations in sample mixtures. The relative integration values, expressed as means are provided in the supplementary data (Tables S5 and S6). Univariate analysis was conducted with MATLAB software and ratio paired t test was applied (Tables S5 and S6). All univariate statistics were considered significant at a p value < 0.05.

### Statistical analysis

Data are shown as mean ± SEM from at least three independent biological experiments. Statistical analyses were performed with the Prism 10.0.3 (GraphPad Software). In most of the analyses, the unpaired two-tailed t test was used, with the exception of analyses of confocal microscopy, GC–MS and ^1^H-NMR experiments in which the ratio paired t test was used. The significance of mean comparison is indicated with ^#^P < 0.1, *P < 0.05, **P < 0.01, ***P < 0.001, ****P < 0.0001. Non-significant results (P > 0.1) were not indicated.

## Supplementary Information

Below is the link to the electronic supplementary material.

Supplementary file1 (PDF 3349 KB)

## Data Availability

The data from microarray analysis have been deposited to ZENODO: 105281zenodo10784726 (https://zenodo.org/records/10784727).
