# Supplementary material for: NFκB and JNK pathways mediate metabolic adaptation upon ESCRT-I deficiency
Source: Cell Mol Life Sci. 2024 Nov 19;81(1):458. doi: 10.1007/s00018-024-05490-y (PMC11576715; doi:10.1007/s00018-024-05490-y)
Supplement: Supplementary file 1 — Supplementary file1 (PDF 3349 KB) [file 18_2024_5490_MOESM1_ESM.pdf]

SUPPLEMENTARY FIGURES AND TABLES FOR:

NFκB and JNK pathways mediate metabolic adaptation upon ESCRT-I deficiency

Jaroslaw Cendrowski[1,*,#], Marta Wrobel[1], Michal Mazur[1], Bartosz Jary[1], Ranjana Maurya[1], Surui Wang[2], Michal Korostynski[3], Anna Dziewulska[4], Maria Rohm[2], Patryk Kuropka[5], Natalia Pudełko-Malik[5], Piotr Młynarz[5], Agnieszka Dobrzyn[4], Anja Zeigerer[2,6] and Marta Miaczynska[1,*]

[1] Laboratory of Cell Biology, International Institute of Molecular and Cell Biology, Warsaw, Poland

[2] Institute for Diabetes and Cancer, Helmholtz Munich, Neuherberg, Germany; Joint Heidelberg-IDC Translational Diabetes Program, Inner Medicine 1, University Hospital, Heidelberg, Germany; German Center for Diabetes Research, Neuherberg, Germany

[3] Laboratory of Pharmacogenomics, Department of Molecular Neuropharmacology, Institute of Pharmacology Polish Academy of Sciences, Krakow, Poland

[4] Laboratory of Cell Signaling and Metabolic Disorders, Nencki Institute of Experimental Biology, Warsaw, Poland

[5] Department of Biochemistry, Molecular Biology and Biotechnology, Faculty of Chemistry, Wroclaw University of Science and Technology, Wroclaw, Poland

[6] European Center for Angioscience (ECAS), Medical Faculty Mannheim, Heidelberg University, Mannheim, Germany

[#] Present address: Department of Genetics, Maria Sklodowska-Curie National Research Institute of Oncology, Warsaw, Poland.

* Corresponding authors.
Tel: +48 22 597 07 24; e-mail: jaroslaw.cendrowski@nio.gov.pl
Tel: +48 22 597 07 25; e-mail: miaczynska@iimcb.gov.pl

SUPPLEMENTARY FIGURE LEGENDS AND FIGURES

Figure S1. ESCRT-I dysfunction leads to reduced expression of genes involved in oxidative metabolism of amino acids and fatty acids in HepG2 cells. (A) qPCR results showing the expression of genes encoding TSG101, VPS28 and the indicated oxidative metabolism enzymes in HepG2 cells with removal of ESCRT-I using single siRNA for each component (siTSG101#2, siVPS28#1), as compared to control cells (treated with non-targeting siRNAs, Ctrl#2 or #3) presented as fold changes with respect to average values for control cells. Mean values (n = 3 ± SEM) are presented. Statistical significance tested by comparison to siCtrl#2. **(B)** Western blots showing the efficiency of CRISPR-Cas9-mediated depletion of TSG101 (using single gRNA) and its effect on the levels of VPS28 protein, as compared to control conditions (non-targeting gRNAs, gCtrl#1 or #2), in cells. Vinculin used as a gel loading control. **(C)** qPCR results showing the expression of genes encoding the indicated oxidative metabolism enzymes in cells with CRISPR-Cas9-mediated depletion of TSG101, as compared to control cells presented as fold changes with respect to average values for control cells. Mean values (n = 4 ± SEM) are presented. Statistical significance tested by comparison to gCtrl#2. *P < 0.05, **P < 0.01, ***P < 0.001.

Figure S2. Expression of several genes encoding enzymes involved in biosynthesis of fatty acid-containing lipids is increased in cells lacking ESCRT-I. (A) Heatmaps visualizing microarray results regarding the expression of genes encoding enzymes of fatty acid (FA), triglyceride (TG) or phospholipid (PL) biosynthesis in HEK293 cells after removal of ESCRT-I using two siRNAs for each component (siTSG101#1 or siTSG101#2, siVPS28#1 or siVPS28#2), as compared to control cells (treated with non-targeting siRNAs, Ctrl#1 or #2). Microarray data analysis was performed based on three independent experiments at three days post transfection with siRNAs (3 dpt). **(B-C)** Dot plots showing number (in B) and mean area (in C) of lipid droplets (LDs) obtained by quantitative analysis of BODIPY 493/503 staining of control cells (treated with two non-targeting siRNAs, Ctrl#2 or #3) or ESCRT-I-deficient cells (treated with siTSG101#2 or siV2PS8#2) shown in Fig. 2D. siCtrl average represents averaged values measured for cells transfected with siCtrl#2 or siCtrl#3. Values derived from independent experiments (dots) and their means (n = 3 ± SEM) are presented. Average number of cells analyzed per condition was 2999 for siCtrl#2, 2758 for siCtrl#3, 1814 for siTSG101#2 and 2202 for siVPS28#2. Statistical significance tested by comparison to siCtrl average values. *P < 0.05, **P < 0.01.

Figure S3. Despite the reduced expression of many genes encoding mitochondrial proteins, cells lacking ESCRT-I do not have lower mitochondria abundance. (A) Gene ontology (GO) analysis of top cellular compartments identified by annotation of genes detected in microarray experiments as those with strongly downregulated expression (log2 fold change ≤ -0.6; FDR < 0.05) in HEK293 cells after removal of ESCRT-I using two siRNAs for each component (siTSG101#1 or siTSG101#2, siVPS28#1 or siVPS28#2), as compared to control cells (treated with non-targeting siRNAs, Ctrl#1 or #2). Microarray data analysis was performed based on three independent experiments at three days post transfection with siRNAs (3 dpt). (**B**) Maximum intensity projection confocal images of fixed control or ESCRT-I-depleted cells stained using antibodies recognizing TOM20 protein (red) or mono- and polyubiquitinated protein conjugates (Ub; green). Cell nuclei marked with DAPI stain (blue). Scale bar, 50 µm. The dot plots show total fluorescence intensities per cell (expressed in arbitrary units, a.u.), as compared to averaged values measured for cells transfected with siCtrl#2 or siCtrl#3 (siCtrl average). Values derived from independent experiments (dots) and their means (n = 3 ± SEM) are presented. Average number of cells analyzed per condition was 1304 for siCtrl#2, 1132 for siCtrl#3, 598 for

siTSG101#2 and 759 for siVPS28#2. Statistical significance tested by comparison to the siCtrl average values. *P < 0.05.

Figure S4. ESCRT-I dysfunction leads to increased expression of genes involved in glycolytic metabolism. (A) Heatmap visualizing microarray results regarding the expression of genes encoding enzymes involved in production of acetyl-CoA from pyruvate in HEK293 cells after removal of ESCRT-I using two siRNAs for each component (siTSG101#1 or siTSG101#2, siVPS28#1 or siVPS28#2), as compared to control cells (treated with non-targeting siRNAs, Ctrl#1 or #2). Microarray data analysis was performed based on three independent experiments at three days post transfection with siRNAs (3 dpt). **(B)** qPCR results showing the expression of genes encoding the indicated glycolytic enzymes at 3 dpt in cells with ESCRT-I removal, as compared to control HEK293 cells. The mean values (n = 4 ± SEM) are presented as fold changes with respect to average values for control cells (siCtrl average). **(C-D)** qPCR results showing the expression of genes encoding the indicated glycolytic metabolism enzymes in HepG2 cells with siRNA-mediated removal (3 dpt) of ESCRT-I components using single siRNA for each component (siTSG101#2, siVPS28#1 in C) or in cells with CRISPR-Cas9-mediated depletion of TSG101 using single gRNA (gTsg101 in D), as compared to respective control cells (treated with non-targeting siRNAs, Ctrl#2 or #3 or gRNAs, gCtrl#1 or #2). The results presented as fold changes with respect to average values for control cells. Mean values (n = 3 ± SEM). Statistical significance tested by comparison to siCtrl average (in B), siCtrl#2 (in C) or gCtrl#1 (in D). $^{\#}$P<0.1, *P < 0.05, **P < 0.01, ***P < 0.001, ****P < 0.0001.

Figure S5. ^{1}H-NMR analysis allowed to detect and quantify the abundance of various extracellular and intracellular metabolites of control or ESCRT-I deficient cells. (A) Representative ^{1}H-NMR spectrum for metabolites detected in extracts from medium collected from HEK293 cells at four days post transfection with siRNAs (4 dpt). The cells were cultured in the medium for 24 h. 1 – Leucine, 2 – Isoleucine, 3 – Valine, 4 – 3-Hydroxyisobutyrate, 5 – 3-Methyl-2-oxovalerate, 6 – Unknown_1, 7 – Unknown_2, 8 – Unknown_3, 9 – Alanine, 10 – Acetate, 11 – Glutamate, 12 – Pyruvate, 13 – Succinate, 14 – Pyroglutamate, 15 – Glutamine, 16 – Methionine, 17 – Citrate, 18 – Lysine, 19 – Arginine, 20 – Choline, 21 – Proline, 22 – Methanol, 23 – Glucose, 24 – Glycine, 25 – Threonine, 26 – Glycerol, 27 – Creatine, 28 – Serine, 29 – Fructose, 30 – myo-Inositol, 31 – Lactate, 32 – Fumarate, 33 – Tyrosine, 34 – Unknown_4, 35 – Phenylalanine, 36 – Unknown_5, 37 – Formate. **(B)** Representative ^{1}H-NMR spectrum for metabolites detected in extracts from pellets of cells collected at 4 dpt. 1 – Leucine, 2 – Isoleucine, 3 – Valine, 4 – Lactate, 5 – Alanine, 6 – Acetate, 7 – Glutamate, 8 – Succinate, 9 – Glutamine, 10 – Citrate, 11 – Glutathione, 12 – Creatine phosphate, 13 – Choline, 14 – o-Phosphocholine, 15 – Methanol, 16 – Taurine, 17 – Glycine, 18 – Threonine, 19 – myo-Inositol, 20 – Serine, 21 – Creatine, 22 – ATP, 23 – ADP. **(C)** Abundance of the indicated BCCAs in the medium from control or ESCRT-I-deficient cells as compared to levels in fresh DMEM (n = 4 ± SEM). **(D-F)** Intracellular levels of BCAAs (in D), ATP and ADP (in E) or glutathione (in F) detected by NMR analysis of metabolites in pellets of control or ESCRT-I-deficient cells (n = 4 ± SEM). *P < 0.05.

Figure S6. mTORC1 signaling does not contribute to reduced expression of genes encoding enzymes of amino acid or fatty acid oxidation upon ESCRT-I deficiency. (A) Representative western blots showing the levels of TSG101 and VPS28 proteins as well as phosphorylated S6 protein (P-S6) in control HEK293 cells (siCtrl#1 or siCtrl#2) or cells depleted of ESCRT-I components (siTSG101#2 or siVPS28#2), treated with DMSO or 100 nM mTOR kinase inhibitor (INK128) for 48 h. The graph (right panel) shows protein levels as fold change with respect to averaged values measured for siCtrl#1 and #2 (siCtrl average) assessed by densitometry analysis of western blotting bands. The analysis was performed at

3 dpt with vinculin used as a gel loading control. **(B-D)** qPCR results showing the expression of genes encoding components of ESCRT-I (in B), TFEB/TFE3 transcription factors (in C) or the indicated enzymes of amino acid or fatty acid oxidation (in D) in control (siCtrl#1) or ESCRT-I-deficient (siTSG101#2 or siVPS28#2) cells with single depletion or with co-depletion of TFEB and TFE3 (siTFEB/TFE3). The results presented as fold changes with respect to siCtrl#1 at 4 dpt. Mean values (n = 4 ± SEM) are presented. Statistical significance in A and C tested by comparison of indicated pairs, whereas in B and D tested by comparison of control cells or cells lacking only TSG101 or VPS28 to respective cells lacking additionally TFEB and TFE3. ^{ns}P> 0.1, $^{\#}$P<0.1, *P < 0.05, **P < 0.01.

Figure S7. Simultaneous inhibition of NFκB or JNK signaling does not interfere with efficient depletion of ESCRT-I components. (A-D) qPCR results showing the efficiencies of silencing the expression of genes encoding the indicated proteins in cells treated with siRNAs targeting the ESCRT-I components (siTSG101#2 or siVPS28#2) or regulators of NFκB signaling (siIKKA in A-B or siRELA in C-D) as compared to control cells (siCtrl#2) at four days post siRNA transfection (4 dpt). Mean values (n = 4 ± SEM in A or n = 3 ± SEM in B) are presented. **(E-H)** qPCR results showing the efficiencies of silencing the expression of genes encoding TSG101 or VPS28 proteins and the levels of IL-8 mRNA in cells treated with siTSG101#2 or siVPS28#2 as compared to controls cells (siCtrl#2) at 4 dpt, after 72 h treatment with DMSO or 50 µM SP600125 compound (in E and G) or 2 µM JNK-IN-8 compound (in F and H). The results presented as fold changes with respect to siCtrl#2. Mean values (n = 3 ± SEM) are presented. Statistical significance in B and D tested by comparison of indicated pairs, whereas in A, C and E-H by comparing the results for siCtrl#2, siTSG101#2 or siVPS28#2, with IKKA/RELA co-depletion or inhibitor treatment with the results for respective cells with single depletions or DMSO-treatment. ^{ns}P> 0.1, $^{\#}$P < 0.1, *P < 0.05, **P < 0.01, ***P < 0.001, ****P < 0.0001.

Figure S8. The effects ESCRT inactivation on ubiquitin accumulation, intracellular signaling and metabolic gene expression. (A) Western blots showing the levels of ESCRT-I components as well as mono- and polyubiquitinated proteins (Ub) in HEK293 cells depleted of ESCRT-I using two single siRNAs for each component (siTSG101#1 or siTSG101#2, siVPS28#1 or siVPS28#2), as compared to control cells (treated with non-targeting siRNAs, Ctrl#1 or #2). The analysis was performed at three days post siRNA transfection (3 dpt) with tubulin used as a gel loading control. **(B)** Graphs representing the results of densitometric analysis of western blots shown in Fig. 7A concerning proteins in cells after siRNA-mediated removal of ESCRT-I, by single depletion (siTSG101#2, siVPS28#2) or co-depletion (siTSG101#2/siVPS28#2) of its components, or removal of ESCRT-III components (siCHMP4B or siCHMP7), as compared to control cells (siCtrl#2). Mean values (n = 3 ± SEM) are presented. **(C)** qPCR results showing the effects of inactivating ESCRT-I, by single depletion (siTSG101#2, siVPS28#2) or co-depletion (siTSG101#2/siVPS28#2) of its components, on the expression of genes encoding the indicated oxidative (top) or glycolytic (bottom) enzymes compared to control (siCtrl#2) cells. Mean values (n = 3 ± SEM) are presented. **(D)** Western blots showing the levels of indicated ESCRT-I components as well as mono- and polyubiquitinated proteins (Ub) in cells after siRNA-mediated removal of TSG101 or VPS28 (at 4 dpt), treated for 24 h with DMSO or 20 nM BafA1, as compared to control cells (treated with non-targeting siRNA, Ctrl#2 and DMSO). Statistical significance in B and C tested by comparison to siCtrl#2. $^{\#}$P < 0.1, *P < 0.05, **P < 0.01, ***P < 0.001, ****P < 0.0001.

Figure S1

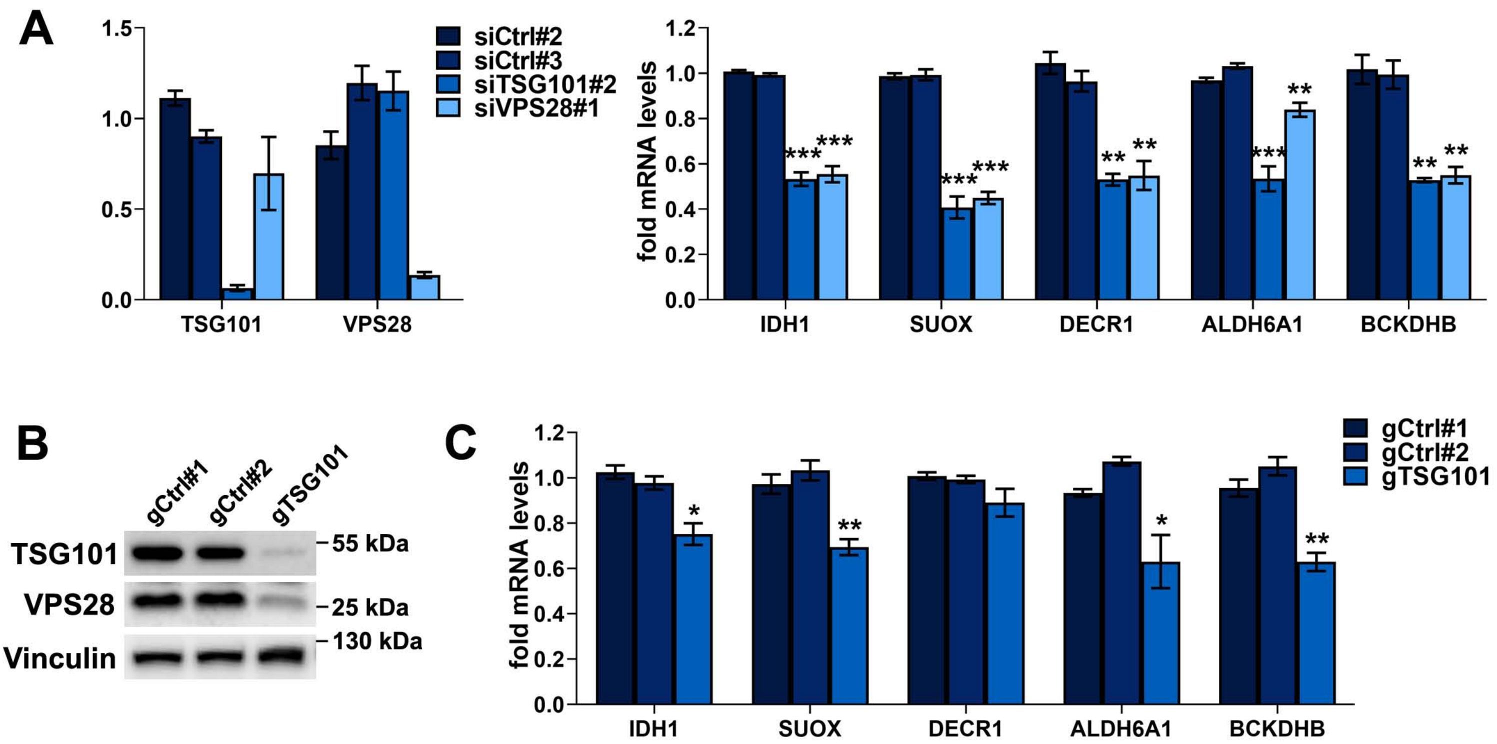

A
1.5
1.0
0.5
0.0
TSG101
VPS28
siCtrl#2
siCtrl#3
siTSG101#2
siVPS28#1
1.2
1.0
0.8
0.6
0.4
0.2
0.0
fold mRNA levels
***
**
*
IDH1
SUOX
DECR1
ALDH6A1
BCKDHB
B
gCtrl#1
gCtrl#2
gTSG101
TSG101
VPS28
Vinculin
55 kDa
25 kDa
130 kDa
C
gCtrl#1
gCtrl#2
gTSG101

Figure S2

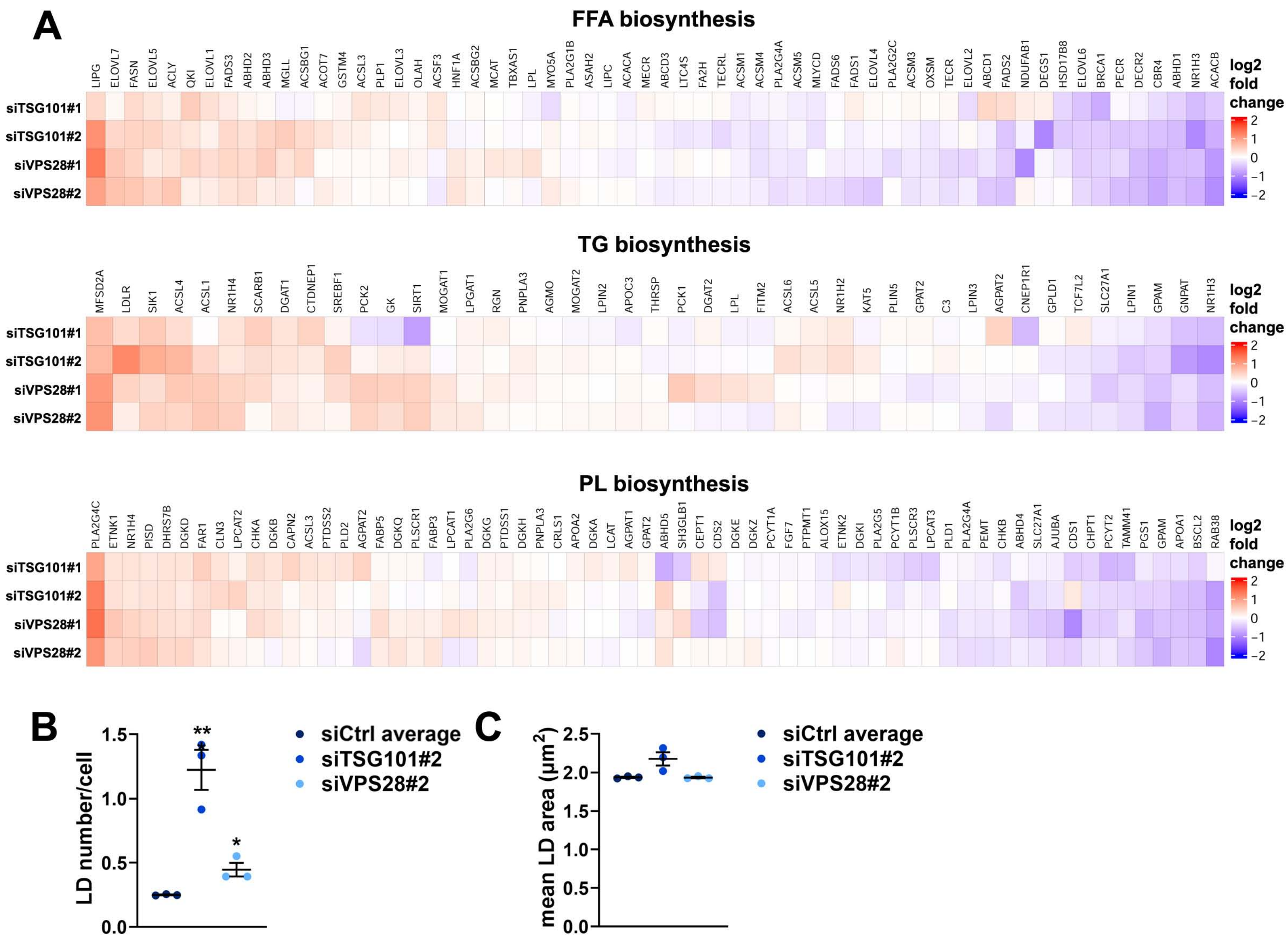
A
FFA biosynthesis
LIPG ELOVL7 FASN ELOVL5 ACLY QKI ELOVL1 FADS3 ABHD2 ABHD3 MGLL ACSBG1 ACOT7 GSTM4 ACSL3 PLP1 ELOVL3 OLAH ACSF3 HNF1A ACSBG2 MCAT TBXAS1 LPL MYO5A PLA2G1B ASAH2 LIPC ACACA MECR ABCD3 LTC4S FA2H TECRL ACSM1 ACSM4 PLA2G4A ACSM5 MLYCD FADS6 FADS1 ELOVL4 PLA2G2C ACSM3 OXSM TECR ELOVL2 ABCD1 FADS2 NDUFAB1 DEGS1 HSD17B8 ELOVL6 BRCA1 PECR DECR2 CBR4 ABHD1 NR1H3 ACACB
siTSG101#1
siTSG101#2
siVPS28#1
siVPS28#2
log2 fold change
2
1
0
−1
−2
TG biosynthesis
MFSD2A LDLR SIK1 ACSL4 ACSL1 NR1H4 SCARB1 DGAT1 CTDNEP1 SREBF1 PCK2 GK SIRT1 MOGAT1 LPGAT1 RGN PNPLA3 AGMO MOGAT2 LPIN2 APOC3 THRSP PCK1 DGAT2 LPL FITM2 ACSL6 ACSL5 NR1H2 KAT5 PLIN5 GPAT2 C3 LPIN3 AGPAT2 CNEP1R1 GPLD1 TCF7L2 SLC27A1 LPIN1 GPAM GNPAT NR1H3
siTSG101#1
siTSG101#2
siVPS28#1
siVPS28#2
log2 fold change
2
1
0
−1
−2
PL biosynthesis
PLA2G4C ETNK1 NR1H4 PISD DHRS7B DGKD FAR1 CLN3 LPCAT2 CHKA DGKB CAPN2 ACSL3 PTDSS2 PLD2 AGPAT2 FABP5 DGKQ PLSCR1 FABP3 LPCAT1 PLA2G6 DGKG PTDSS1 DGKH PNPLA3 CRLS1 APOA2 DGKA LCAT AGPAT1 GPAT2 ABHD5 SH3GLB1 CEPT1 CDS2 DGKE DGKZ PCYT1A FGF7 PTPMT1 ALOX15 ETNK2 DGKI PLA2G5 PCYT1B PLSCR3 LPCAT3 PLD1 PLA2G4A PEMT CHKB ABHD4 SLC27A1 AJUBA CDS1 CHPT1 PCYT2 TAMM41 PGS1 GPAM APOA1 BSCL2 RAB38
siTSG101#1
siTSG101#2
siVPS28#1
siVPS28#2
log2 fold change
2
1
0
−1
−2
B
LD number/cell
1.5
1.0
0.5
0.0
**
*
siCtrl average
siTSG101#2
siVPS28#2
C
mean LD area (µm²)
2.5
2.0
1.5
1.0
0.5
0.0
siCtrl average
siTSG101#2
siVPS28#2

Figure S3

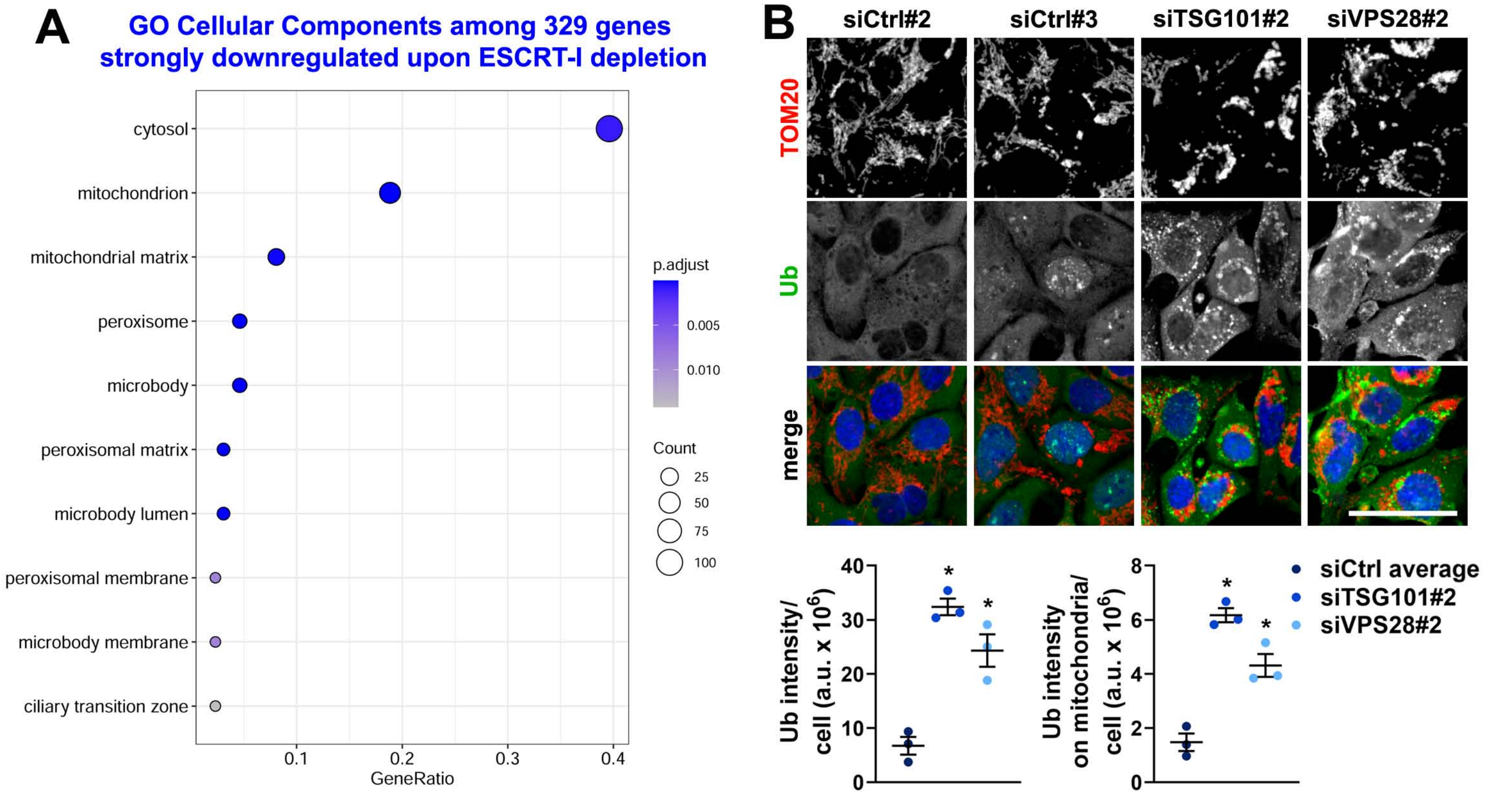
A
GO Cellular Components among 329 genes strongly downregulated upon ESCRT-I depletion
cytosol
mitochondrion
mitochondrial matrix
peroxisome
microbody
peroxisomal matrix
microbody lumen
peroxisomal membrane
microbody membrane
ciliary transition zone
0.1
0.2
0.3
0.4
GeneRatio
p.adjust
0.005
0.010
Count
25
50
75
100
B
siCtrl#2
siCtrl#3
siTSG101#2
siVPS28#2
TOM20
Ub
merge
Ub intensity/ cell (a.u. x 10^6)
Ub intensity on mitochondria/ cell (a.u. x 10^6)
siCtrl average
siTSG101#2
siVPS28#2

Figure S4

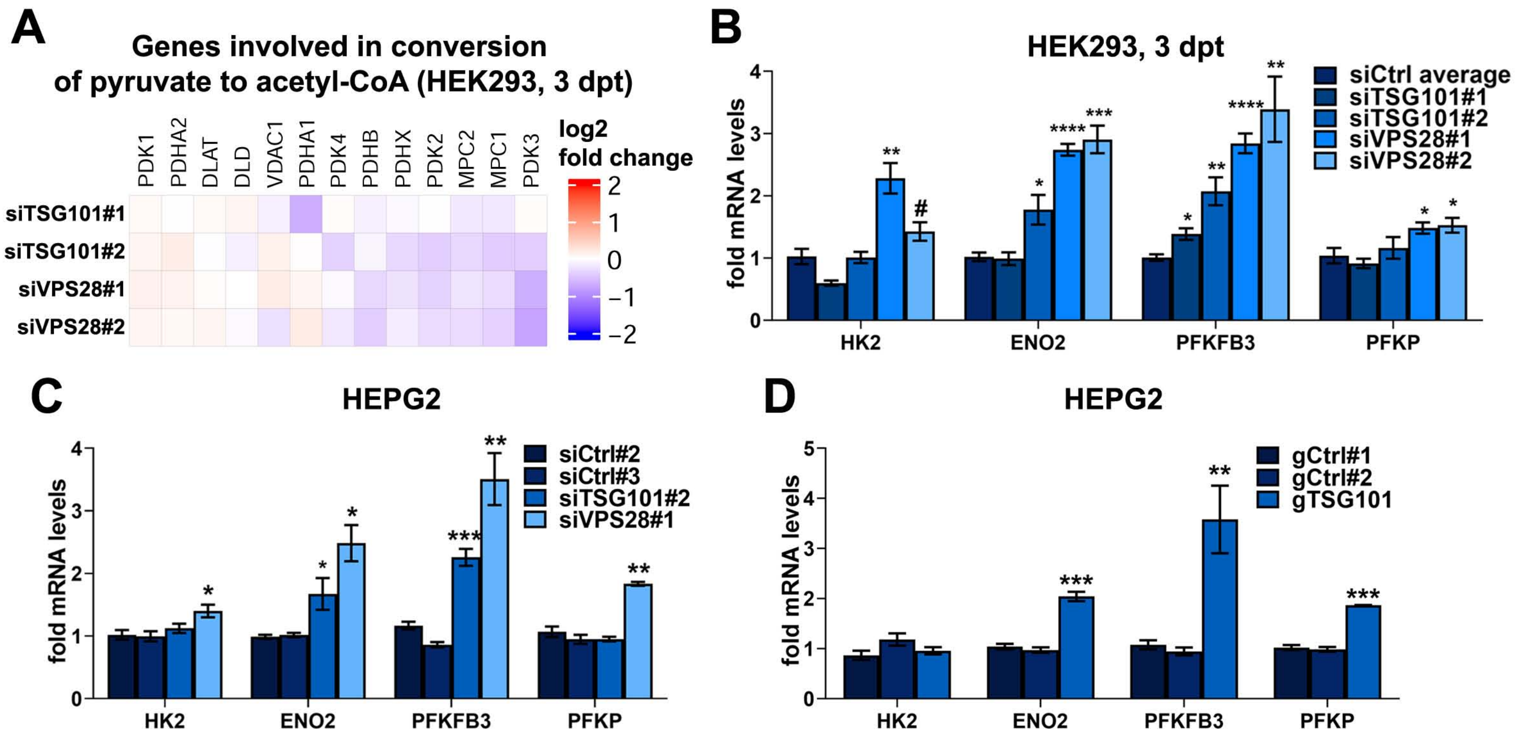
A
Genes involved in conversion of pyruvate to acetyl-CoA (HEK293, 3 dpt)
PDK1 PDHA2 DLAT DLD VDAC1 PDHA1 PDK4 PDHB PDHX PDK2 MPC2 MPC1 PDK3
siTSG101#1
siTSG101#2
siVPS28#1
siVPS28#2
log2 fold change
2 1 0 −1 −2
B
HEK293, 3 dpt
fold mRNA levels
siCtrl average
siTSG101#1
siTSG101#2
siVPS28#1
siVPS28#2
HK2
ENO2
PFKFB3
PFKP
C
HEPG2
fold mRNA levels
siCtrl#2
siCtrl#3
siTSG101#2
siVPS28#1
HK2
ENO2
PFKFB3
PFKP
D
HEPG2
fold mRNA levels
gCtrl#1
gCtrl#2
gTSG101
HK2
ENO2
PFKFB3
PFKP

Figure S5

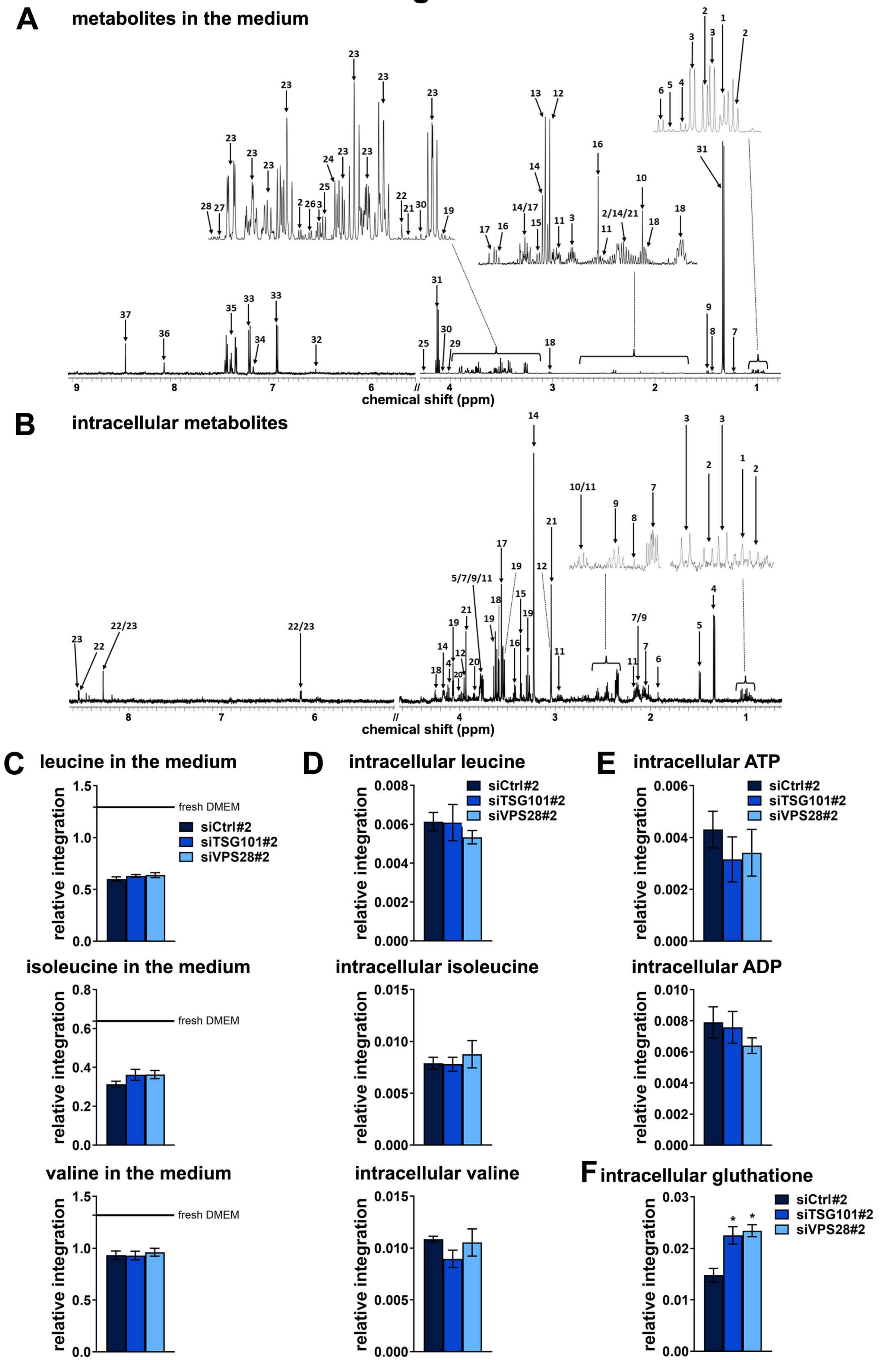
A
metabolites in the medium
chemical shift (ppm)
B
intracellular metabolites
chemical shift (ppm)
C
leucine in the medium
isoleucine in the medium
valine in the medium
fresh DMEM
relative integration
siCtrl#2
siTSG101#2
siVPS28#2
D
intracellular leucine
intracellular isoleucine
intracellular valine
E
intracellular ATP
intracellular ADP
F
intracellular gluthatione

Figure S6

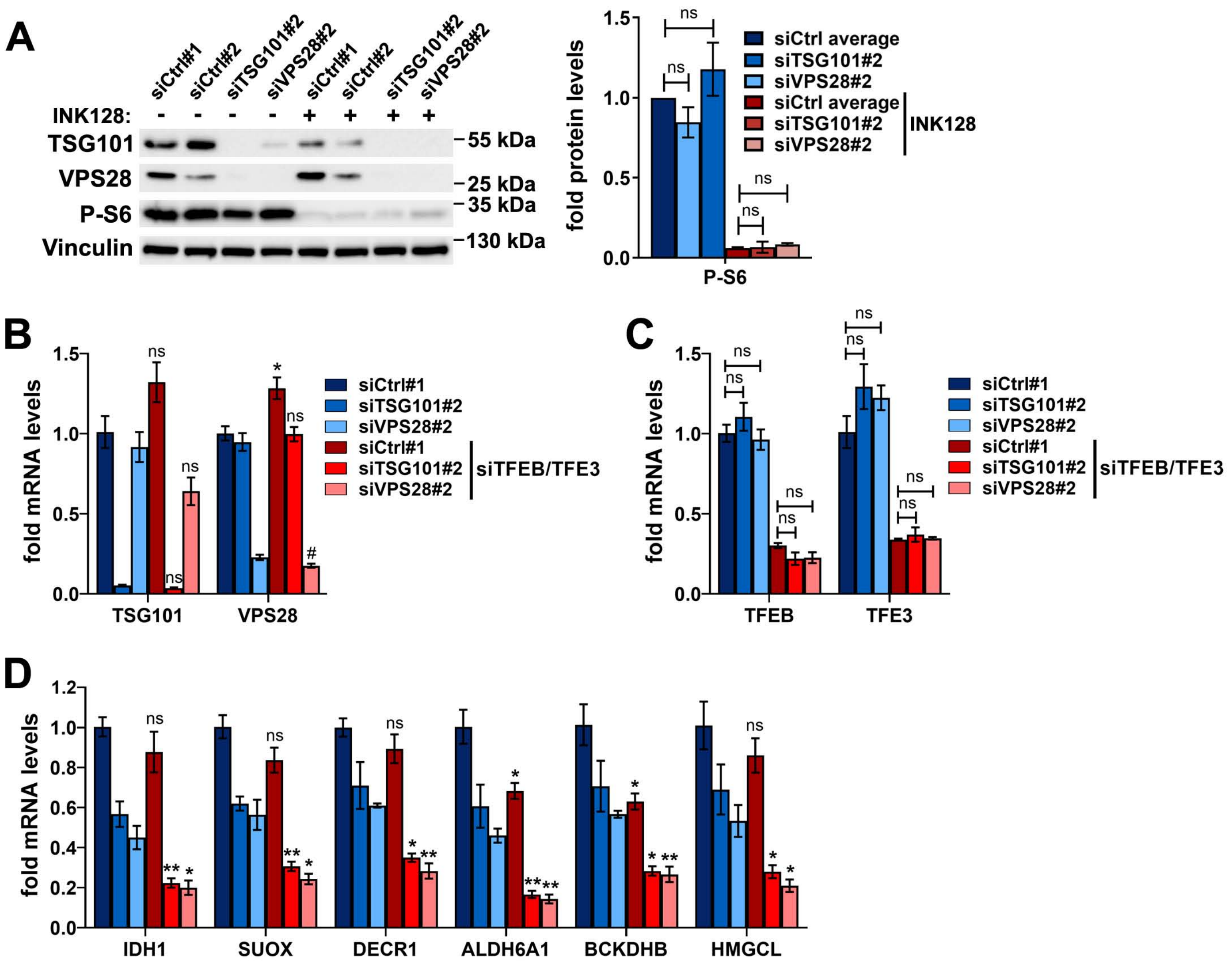
A
siCtrl#1
siCtrl#2
siTSG101#2
siVPS28#2
siCtrl#1
siCtrl#2
siTSG101#2
siVPS28#2
INK128: - - - - + + + +
TSG101
VPS28
P-S6
Vinculin
55 kDa
25 kDa
35 kDa
130 kDa
fold protein levels
siCtrl average
siTSG101#2
siVPS28#2
siCtrl average
siTSG101#2
siVPS28#2
INK128
ns
P-S6
B
fold mRNA levels
siCtrl#1
siTSG101#2
siVPS28#2
siCtrl#1
siTSG101#2
siVPS28#2
siTFEB/TFE3
TSG101
VPS28
ns
*
#
C
fold mRNA levels
siCtrl#1
siTSG101#2
siVPS28#2
siCtrl#1
siTSG101#2
siVPS28#2
siTFEB/TFE3
TFEB
TFE3
ns
D
fold mRNA levels
IDH1
SUOX
DECR1
ALDH6A1
BCKDHB
HMGCL
ns
*
**

Figure S7

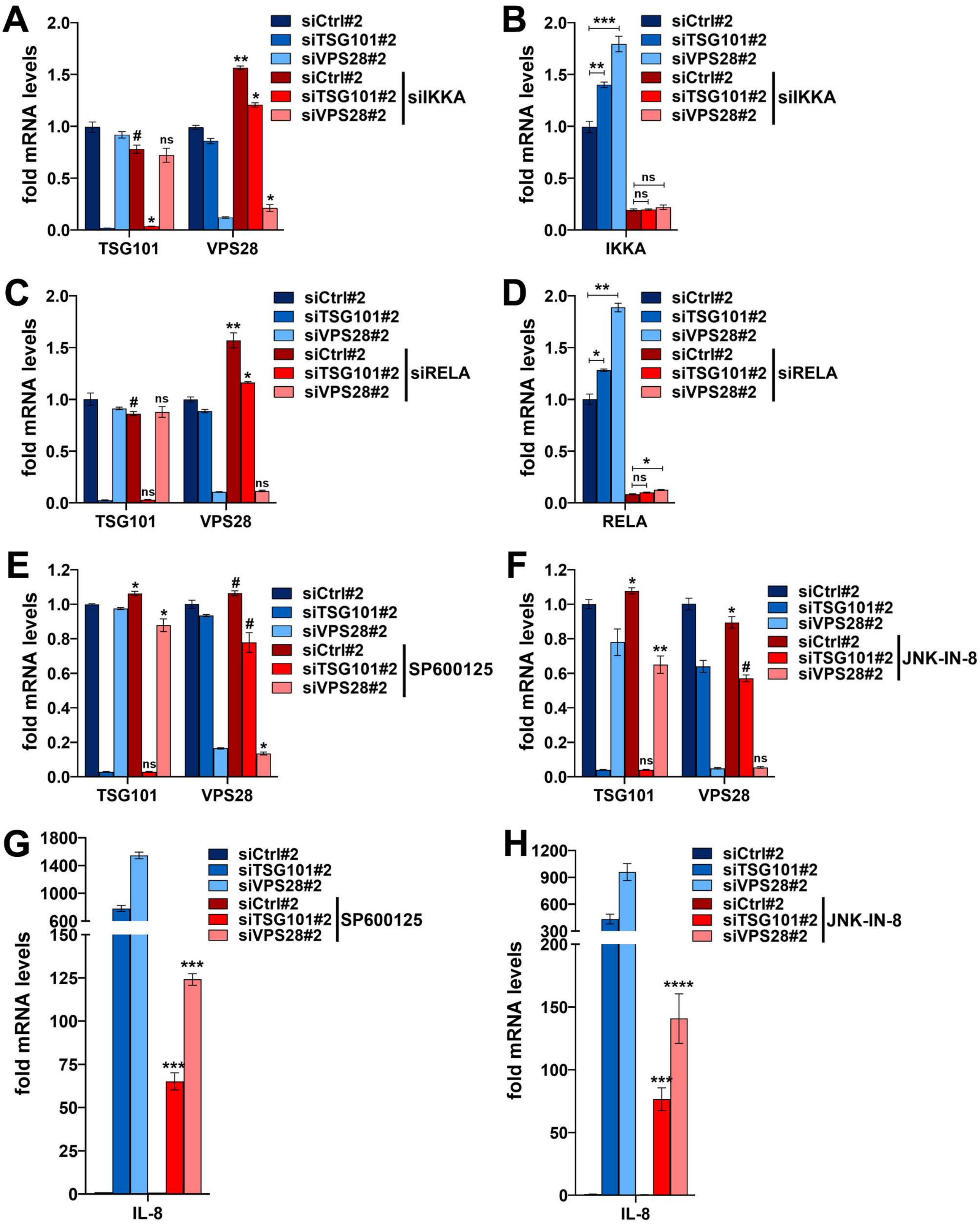

A
fold mRNA levels
TSG101
VPS28
siCtrl#2
siTSG101#2
siVPS28#2
siCtrl#2
siTSG101#2
siVPS28#2
siIKKA
B
IKKA
C
siRELA
D
RELA
E
SP600125
F
JNK-IN-8
G
IL-8
H

Figure S8

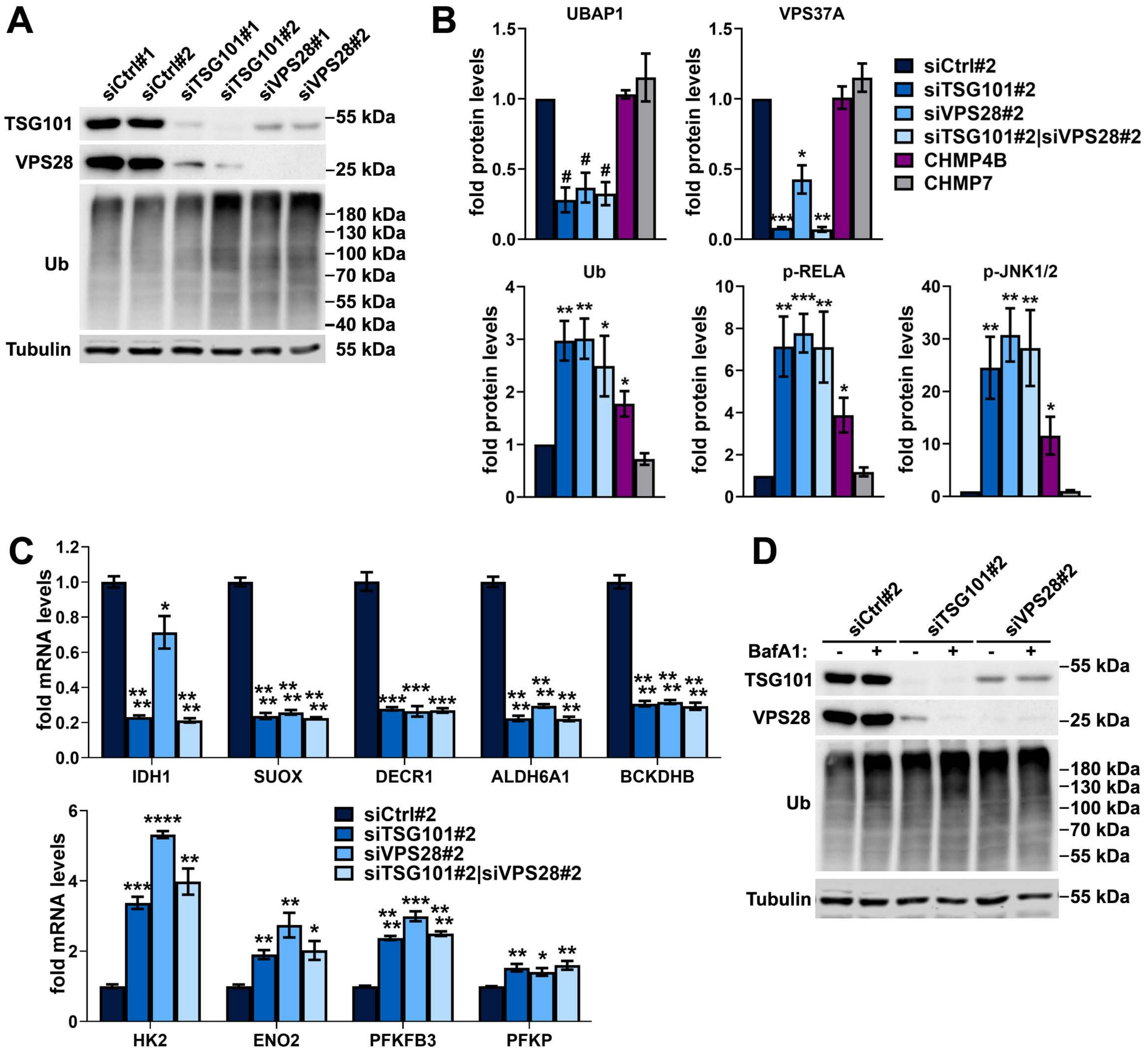
A
siCtrl#1
siCtrl#2
siTSG101#1
siTSG101#2
siVPS28#1
siVPS28#2
TSG101
VPS28
Ub
Tubulin
55 kDa
25 kDa
180 kDa
130 kDa
100 kDa
70 kDa
55 kDa
40 kDa
55 kDa
B
UBAP1
VPS37A
Ub
p-RELA
p-JNK1/2
fold protein levels
siCtrl#2
siTSG101#2
siVPS28#2
siTSG101#2|siVPS28#2
CHMP4B
CHMP7
C
fold mRNA levels
IDH1
SUOX
DECR1
ALDH6A1
BCKDHB
HK2
ENO2
PFKFB3
PFKP
siCtrl#2
siTSG101#2
siVPS28#2
siTSG101#2|siVPS28#2
D
siCtrl#2
siTSG101#2
siVPS28#2
BafA1:
TSG101
VPS28
Ub
Tubulin
55 kDa
25 kDa
180 kDa
130 kDa
100 kDa
70 kDa
55 kDa
55 kDa

SUPPLEMENTARY TABLES

Table S1. List of genes downregulated upon ESCRT-I depletion annotated to small molecule metabolic process. Indicated are average fold changes, false discovery rates (FDR) and information whether each gene is involved in amino acid or fatty acid oxidation or in other process category.

**GO:0044281: small molecule metabolic process**			
**Gene**	**Average log2 fold change**	**FDR**	**Category**
NAALAD2	-1.1465	0.0007	amino acid oxidation
SUOX	-1.1192	0.0318	amino acid oxidation
NME3	-1.1189	0.0079	other
COQ5	-1.0477	0.0208	other
OARD1	-1.0106	0.0013	other
CACNA1H	-0.9718	0.0053	other
HOGA1	-0.9714	0.0028	amino acid oxidation
TM7SF2	-0.9179	0.0107	other
IDH1	-0.9109	0.0076	fatty acid oxidation
EPHX2	-0.8995	0.0065	fatty acid oxidation
ALDH3A2	-0.8976	0.0050	fatty acid oxidation
IDNK	-0.8878	0.0121	other
CLYBL	-0.8567	0.0121	other
NUDT7	-0.8554	0.0037	fatty acid oxidation
ACCS	-0.8552	0.0330	other
CYP4V2	-0.8495	0.0165	other
GSTM2	-0.8487	0.0494	other
BPHL	-0.8487	0.0147	other
SIRT5	-0.7967	0.0002	other
MIDN	-0.7699	0.0085	other
PLCB1	-0.7625	0.0123	fatty acid oxidation
NNT	-0.7586	0.0141	other
PEX2	-0.7578	0.0054	fatty acid oxidation
ACACB	-0.7557	0.0336	fatty acid oxidation
UQCR10	-0.7429	0.0173	other
SUCLG2	-0.7360	0.0097	other
ATP2B4	-0.7351	0.0439	amino acid oxidation
NR1H3	-0.7113	0.0114	other
BCKDHB	-0.7098	0.0013	amino acid oxidation
ALDH7A1	-0.7096	0.0015	amino acid oxidation
ATP6V1B1	-0.7087	0.0139	other
HMGCL	-0.7057	0.0040	amino acid oxidation
CPT2	-0.6978	0.0127	fatty acid oxidation
EXTL2	-0.6928	0.0057	other
DECR1	-0.6877	0.0182	fatty acid oxidation
PEX7	-0.6849	0.0027	fatty acid oxidation
CSAD	-0.6772	0.0028	amino acid oxidation
GALM	-0.6737	0.0010	other
LDHC	-0.6736	0.0035	other

TRERF1	-0.6684	0.0094	fatty acid oxidation
THEM4	-0.6629	0.0173	fatty acid oxidation
TST	-0.6583	0.0049	amino acid oxidation
MST1	-0.6501	0.0215	other
RDH10	-0.6488	0.0070	other
ECI2	-0.6340	0.0005	fatty acid oxidation
DCAKD	-0.6336	0.0053	other
CAV1	-0.6276	0.0300	other
CRYZL1	-0.6245	0.0334	other
ALDH6A1	-0.6178	0.0154	amino acid oxidation
CAT	-0.6172	0.0294	other
PPA2	-0.6171	0.0068	other
ACSF2	-0.6143	0.0083	fatty acid oxidation
UROS	-0.6115	0.0166	other
DDIT4	-0.6052	0.0315	other
NUDT1	-0.6044	0.0101	other
PLA2G4B	-0.6028	0.0049	fatty acid oxidation
EHHADH	-0.6022	0.0042	fatty acid oxidation

Table S2. List of genes downregulated upon ESCRT-I depletion annotated to mitochondrion. Indicated are average fold changes, false discovery rates (FDR) and information whether each gene is involved in amino acid or fatty acid oxidation or in other process category.

**GO:0005739: mitochondrion**			
**Gene**	**Average log2 fold change**	**FDR**	**Category**
SUOX	-1.1192	0.0318	amino acid oxidation
MSRB2	-1.0815	0.0033	other
COQ5	-1.0477	0.0208	other
NRP1	-1.0194	0.0028	other
BMF	-0.9904	0.0103	other
HOGA1	-0.9714	0.0028	amino acid oxidation
ASB9	-0.9615	0.0180	other
IDH1	-0.9109	0.0076	fatty acid oxidation
CLYBL	-0.8567	0.0121	other
BPHL	-0.8487	0.0147	other
CA5B	-0.8263	0.0249	other
CMC4	-0.8254	0.0107	other
IFI27L1	-0.8239	0.0130	other
PYROXD2	-0.8212	0.0084	other
SIRT5	-0.7967	0.0002	other
NNT	-0.7586	0.0141	other
ACACB	-0.7557	0.0336	fatty acid oxidation
UQCR10	-0.7429	0.0173	other
SUCLG2	-0.7360	0.0097	other
EXD2	-0.7195	0.0149	other
LIPT2	-0.7169	0.0062	other
BCKDHB	-0.7098	0.0013	amino acid oxidation

ALDH7A1	-0.7096	0.0015	amino acid oxidation
HMGCL	-0.7057	0.0040	amino acid oxidation
TMEM143	-0.6984	0.0365	other
CPT2	-0.6978	0.0127	fatty acid oxidation
DECR1	-0.6877	0.0182	fatty acid oxidation
SLC25A42	-0.6787	0.0052	other
LDHC	-0.6736	0.0035	other
THEM4	-0.6629	0.0173	other
TST	-0.6583	0.0049	amino acid oxidation
NFU1	-0.6542	0.0068	other
COA5	-0.6529	0.0479	other
TMEM135	-0.6374	0.0381	other
ECI2	-0.6340	0.0005	fatty acid oxidation
PTCD2	-0.6303	0.0090	other
TXNRD2	-0.6178	0.0028	other
ALDH6A1	-0.6178	0.0154	amino acid oxidation
CAT	-0.6172	0.0294	other
PPA2	-0.6171	0.0068	other
ACSF2	-0.6143	0.0083	fatty acid oxidation
LETMD1	-0.6123	0.0048	other
UROS	-0.6115	0.0166	other
BRD8	-0.6099	0.0080	other
PRIMPOL	-0.6067	0.0080	other
RMDN1	-0.6054	0.0163	other
DDIT4	-0.6052	0.0315	other
NUDT1	-0.6044	0.0101	other
PLA2G4B	-0.6028	0.0049	fatty acid oxidation

Table S3. NMR assignments of metabolites detected by ^{1}H-NMR in medium samples, shown with chemical shifts, number of detected peaks (multiplicity) and HMDBI IDs.

	Metabolite	Chemical shift [ppm]	Multiplicity	HMDB ID
1	Leucine	0.963	t	HMDB00687
2	Isoleucine	1.013	d	HMDB00172
3	Valine	1.044	d	HMDB0000883
4	3-hydroxyisobutyrate	1.068	d	HMDB0000023/ HMDB0000336
5	3-methyl-2-oxovalerate	1.098	d	HMDB0000491
6	Unknown_1	1.124	d	-
7	Unknown_2	1.224	d	-
8	Unknown_3	1.437	d	-
9	Alanine	1.487	d	HMDB0000161
10	Acetate	1.924	s	HMDB0000042
11	Glutamate	2.347	m	HMDB0000148
12	Pyruvate	2.383	s	HMDB0000243
13	Succinate	2.405	s	HMDB0000254
14	Pyroglutamate	2.419	t	HMDB0000267
15	Glutamine	2.457	m	HMDB0000641

16	Methionine	2.647	t	HMDB0000696
17	Citrate	2.672	d	HMDB0000094
18	Lysine	3.029	t	HMDB0003405
19	Arginine	3.233	m	HMDB0003416
20	Proline	3.339	m	HMDB0000162
21	Methanol**	3.360	s	HMDB0001875
22	Glucose	3.510	t	HMDB0000122
23	Glycine	3.572	s	HMDB0000123
24	Threonine	3.605	d	HMDB0000167
25	Glycerol	3.650	d	HMDB0000131
26	Creatine	3.937	s	HMDB0000064
27	Serine	3.967	d	HMDB0000187
28	Fructose	4.042	m	HMDB0000660
29	myo-Inositol	4.084	t	HMDB0000211
30	Lactate	4.124	q	HMDB0000190
31	Fumarate	6.522	s	HMDB0000134
32	Tyrosine	6.915	d	HMDB0000158
33	Unknown_4	7.156	m	-
34	Phenylalanine	7.334	d	HMDB0000159
35	Unknown_5	8.060	m	-
36	Formate	8.459	s	HMDB0000142
37	Niacinamide*	8.950	m	HMDB0001406

s – singlet; d – doublet; t – triplet; q-quartet; m – multiplet; * metabolite not subjected to quantitative analysis due to the lack of a quantifiable unique signal; **the signal from extraction solvent, not subjected to quantitative analysis

Table S4. NMR assignments of metabolites detected by ^{1}H-NMR in cell pellet samples, shown with chemical shifts, number of detected peaks (multiplicity) and HMDBI IDs.

	**Metabolite**	**Chemical shift [ppm]**	**Multiplicity**	**HMDB ID**
1	Leucine	0.951	t	HMDB00687
2	Isoleucine	0.999	d	HMDB00172
3	Valine	1.031	d	HMDB0000883
4	Lactate	4.101	q	HMDB0000190
5	Alanine	1.467	d	HMDB0000161
6	Acetate	1.905	s	HMDB0000042
7	Glutamate	2.337	m	HMDB0000148
8	Succinate	2.389	s	HMDB0000254
9	Glutamine	2.441	m	HMDB0000641
10	Citrate*	2.675	d	HMDB0000094
11	Glutathione	2.946	m	HMDB0000125
12	Creatine phosphate	3.938	s	HMDB0001511
13	Choline*	3.191	s	HMDB0000097
14	o-Phosphocholine	3.208	s	HMDB0001565
15	Methanol**	3.346	s	HMDB0001875
16	Taurine	3.409	t	HMDB0000251
17	Glycine	3.548	s	HMDB0000123
18	Threonine	3.576	d	HMDB0000167

19	Myo-inositol	3.611	t	HMDB0000211
20	Serine	3.620	m	HMDB0000187
21	Creatine	3.919	s	HMDB0000064
22	ATP	8.518	s	HMDB0003665
23	ADP	8.525	s	HMDB0001341

s – singlet; d – doublet; t – triplet; q-quartet; m – multiplet; * metabolite not subjected to quantitative analysis due to the lack of a quantifiable unique signal; **the signal from extraction solvent, not subjected to quantitative analysis

Table S5. Relative integration values, expressed as means with standard deviations, and p-values from statistical analyses, calculated for metabolites detected by ^{1}H-NMR in medium samples.

	**Means**	**Means and standard deviations (SD)**						**p-values**	
**Detected Metabolites:**	**Fresh DMEM**	**siCtrl#2**	**SD**	**siTSG101#2**	**SD**	**siVPS28 #2**	**SD**	**siCtrl#2 vs siTSG101#2**	**siCtrl#2 vs siVPS28#2**
Leucine	1.2927	0.6004	0.0421	0.6296	0.0277	0.6378	0.0486	0.2645	0.2589
Isoleucine	0.6385	0.3132	0.0317	0.3614	0.0566	0.3629	0.0420	0.0772	0.2257
Valine	1.3186	0.9331	0.0814	0.9297	0.0821	0.9617	0.0766	0.9192	0.6592
3-hydroxyisobutyrate	0.0042	0.0695	0.0074	0.0609	0.0032	0.0570	0.0058	0.1192	**0.0157**
3-methyl-2-oxovalerate	0.0037	0.0283	0.0066	0.0296	0.0039	0.0266	0.0032	0.7745	0.7964
Unknown_1	0.0055	0.0864	0.0096	0.0776	0.0059	0.0778	0.0085	0.2373	0.3251
Unknown_2	0.0146	0.0682	0.0030	0.0959	0.0032	0.1331	0.0037	**0.0007**	**0.0001**
Unknown_3	0.0106	0.0429	0.0034	0.0611	0.0052	0.0832	0.0133	**0.0053**	**0.0096**
Alanine	0.1540	0.4189	0.0237	0.3178	0.0169	0.3919	0.0357	**0.0021**	**0.0427**
Acetate	0.1487	0.1896	0.0026	0.1828	0.0163	0.1560	0.0085	0.3864	**0.0066**
Glutamate	0.0177	0.0086	0.0014	0.0168	0.0056	0.0268	0.0022	0.0519	**0.0007**
Pyruvate	0.0112	0.6272	0.0462	0.3744	0.0345	0.2921	0.0299	**0.0025**	**0.0015**
Succinate	0.6664	0.4528	0.0885	0.4632	0.0471	0.4581	0.1177	0.5600	0.9691
Pyroglutamate	0.2986	0.1592	0.0538	0.1537	0.0458	0.1270	0.0588	0.3328	**0.0170**
Glutamine	0.1837	0.0967	0.0121	0.0832	0.0116	0.0510	0.0064	**0.0343**	**0.0016**
Methionine	0.0388	0.0336	0.0058	0.0302	0.0045	0.0255	0.0040	**0.0277**	**0.0043**
Citrate	0.0140	0.0292	0.0040	0.0364	0.0067	0.0327	0.0008	0.0597	0.1202
Lysine	0.1737	0.1458	0.0109	0.1358	0.0151	0.1490	0.0061	0.1557	0.7149
Arginine	0.1050	0.0953	0.0132	0.1059	0.0153	0.1118	0.0049	0.1319	0.0982
Glucose	10.6803	7.9497	0.6203	7.0954	0.7323	4.7845	0.4347	**0.0130**	**0.0046**
Proline	0.0029	0.0310	0.0044	0.0307	0.0032	0.0344	0.0059	0.8075	0.4809
Glycine	0.8440	0.7781	0.0370	0.7630	0.0558	0.6835	0.0182	0.4034	**0.0079**
Threonine	0.5542	0.4698	0.0593	0.4664	0.0513	0.4351	0.0222	0.9326	0.1505
Glycerol	0.1630	0.1660	0.0317	0.1911	0.0256	0.2074	0.0144	0.2556	0.1356
Creatine	0.0376	0.0279	0.0043	0.0316	0.0073	0.0467	0.0047	0.3231	**0.0047**
Serine	0.0888	0.0387	0.0100	0.0288	0.0050	0.0236	0.0033	**0.0443**	**0.0426**
Fructose	0.1054	0.0999	0.0078	0.1090	0.0056	0.1062	0.0150	0.1640	0.3915

myo-Inositol	0.0845	0.0922	0.0198	0.1231	0.0148	0.1372	0.0075	0.1482	**0.0259**
Lactate	1.2966	7.8148	0.3052	10.0201	0.7084	13.9659	0.3086	**0.0038**	**0.0003**
Fumarate	0.0022	0.0060	0.0038	0.0062	0.0008	0.0111	0.0020	0.5637	0.1118
Tyrosine	0.4810	0.3822	0.0118	0.3702	0.0314	0.3825	0.0216	0.4730	0.9986
Unknown_4	0.0680	0.0558	0.0031	0.0553	0.0128	0.0501	0.0039	0.8310	0.1030
Phenylalanine	0.5746	0.4063	0.0220	0.3953	0.0190	0.3607	0.0236	0.6208	**0.0044**
Unknown_5	0.0766	0.0666	0.0163	0.0629	0.0099	0.0640	0.0077	0.7643	0.8812
Formate	0.0272	0.0509	0.0039	0.0686	0.0034	0.0496	0.0056	**0.0054**	0.3357

green color indicates significance with $p<0.05$

Table S6. Relative integration values, expressed as means with standard deviations, and p-values from statistical analyses, calculated for metabolites detected by ^{1}H-NMR in cell pellet samples.

	**Means and standard deviations (SD)**						**p-values**	
**Detected Metabolites:**	**siCtrl#2**	**SD**	**siTSG101#2**	**SD**	**siVPS28 #2**	**SD**	**siCtrl#2 vs siTSG101#2**	**siCtrl#2 vs siVPS28#2**
Leucine	0.0061	0.0010	0.0061	0.0019	0.0053	0.0007	0.8519	0.2698
Isoleucine	0.0079	0.0012	0.0078	0.0014	0.0088	0.0026	0.8606	0.6560
Valine	0.0109	0.0005	0.0090	0.0017	0.0105	0.0026	0.1124	0.7232
Lactate	0.0124	0.0031	0.0133	0.0029	0.0161	0.0065	0.4409	0.1475
Alanine	0.1068	0.0145	0.0933	0.0200	0.1071	0.0475	0.1716	0.7807
Acetate	0.0028	0.0006	0.0029	0.0006	0.0045	0.0010	0.9888	**0.0254**
Glutamate	0.0745	0.0086	0.0916	0.0124	0.0539	0.0083	0.0763	**0.0395**
Succinate	0.0032	0.0008	0.0022	0.0008	0.0032	0.0009	**0.0084**	0.9750
Glutamine	0.0395	0.0049	0.0250	0.0044	0.0084	0.0022	**0.0265**	**0.0005**
Glutathione	0.0148	0.0026	0.0225	0.0034	0.0234	0.0023	**0.0398**	**0.0337**
Creatine phosphate	0.0158	0.0021	0.0160	0.0029	0.0075	0.0011	0.8811	**0.0050**
o-Phosphocholine	0.2234	0.0132	0.1726	0.0317	0.1235	0.0245	0.0566	**0.0124**
Taurine	0.0189	0.0020	0.0169	0.0024	0.0098	0.0034	0.3300	**0.0273**
Glycine	0.0308	0.0060	0.0274	0.0039	0.0184	0.0046	0.1516	**0.0154**
Threonine	0.0328	0.0044	0.0287	0.0079	0.0170	0.0042	0.4843	**0.0026**
myo-Inositol	0.0480	0.0041	0.0108	0.0015	0.0119	0.0028	**<0.0001**	**0.0017**
Serine	0.0059	0.0003	0.0040	0.0016	0.0029	0.0019	0.1334	0.0511
Creatine	0.0260	0.0059	0.0276	0.0097	0.0183	0.0046	0.7803	0.0733
ATP	0.0043	0.0014	0.0032	0.0017	0.0034	0.0018	0.0664	0.5323
ADP	0.0079	0.0020	0.0076	0.0021	0.0064	0.0010	0.6342	0.4115

green color indicates significance with $p<0.05$
